# Frontline decision autonomy under decentralization: Evidence from health sector reform

**DOI:** 10.1371/journal.pone.0343736

**Published:** 2026-05-15

**Authors:** Alan Zarychta, Krister Andersson, Thomas Bossert

**Affiliations:** 1 University of Chicago, Chicago, Illinois, United States of America; 2 University of Notre Dame, Notre Dame, Indiana, United States of America; 3 Harvard T.H. Chan School of Public Health, Boston, Massachusetts , United States of America; Universidade de Lisboa Instituto Superior de Ciencias Sociais e Politicas, PORTUGAL

## Abstract

Decentralization is designed to transfer autonomy from more central to more local actors, yet studies show differences in the autonomy realized under these reforms. Drawing on the case of health sector decentralization in Honduras, we utilize the decision space approach and original data for over 600 frontline health workers in a matched sample to explain facility-level autonomy. We find that reported decision autonomy in four functional areas is slightly reduced under decentralization, reductions are most pronounced where decentralization is led by municipal governments or associations, rather than NGOs, and differences across staff types are modest. Furthermore, capacity and resources are necessary for expanded autonomy, particularly in organizing service delivery, while supportive accountability allows for increased autonomy in human resources and finances. Our research shows the importance of including frontline staff in studies of decision autonomy under decentralization and considering the distribution of autonomy across levels in hierarchical service delivery systems.

## Introduction

Decentralization has been one of the most prevalent public sector governance reforms in countries of the Global South over the last four decades [1,2]. Across diverse policy areas, governments have shifted *de jure* authorities, responsibilities, and resources away from national-level organizations and toward regional, municipal, and other more local organizations. This dynamic has been particularly pronounced in health service delivery where the large majority of countries includes some commitment to decentralization in their national health plans [3, p. 200]. Among the several rationales motivating decentralization reform under the auspices of improved responsiveness [4–6], a common idea is that allotting more local officials sufficient autonomy will allow them to be able to take decisions and actions that better align with their communities’ preferences and needs, which will then improve public sector performance.

More recently, additional reforms inspired by the New Public Management (NPM) have been layered on top of pre-existing decentralization or alongside new efforts to decentralize across Latin America [7-9]. Different combinations of performance-based management and contracting-out are now commonplace within decentralized social service systems in health, education, and the environment. For example, in addition to traditional decentralization to municipal governments, non-governmental organizations (NGOs) are increasingly being used as intermediaries in decentralized health service delivery [10,11]. Additionally, financial incentives tied to meeting performance indicators are being employed by national ministries vis-à-vis regional or local organizations that directly produce health services for their populations [12,13]. While these hybrid administrative forms still provide for a degree of autonomy by local actors under decentralization, the dynamics of control may be as prominent as, or potentially even more so, than decision autonomy [14–16].

Acknowledging enduring debates about conceptualizing decentralization [17,18], one major presumption across these reforms is that local actors will in fact have a meaningful degree of decision autonomy in at least some aspects of public service delivery. Otherwise, some argue, decentralization cannot be said to have occurred [19]. While this is true in principle, three dynamics serve to complicate this relationship, particularly in the context of contemporary administrative reform within Latin America. First, the decentralization literature has shown that *de jure* decision autonomy does not translate seamlessly to *defacto* decision autonomy; just because autonomy is granted in law or policy, does not necessarily mean it can or will be enacted in practice [20–22]. Instead, recent research has focused on identifying the local conditions that can facilitate or impede the exercise of autonomy, and related notions of discretion, in local decision-making [23–25]. Second, within public administration, the policy implementation literature has emphasized the important role of street-level bureaucrats (SLBs), sometimes also called frontline service providers, in translating national-level policy mandates into the decisions and activities experienced by individuals at the local level [26,27]. Research on decentralization has generally emphasized structural explanations at the system or organization level, and only very recently considered the behavior of SLBs, particularly autonomy or discretion, as a key factor that can vary under a common reform and potentially influence service delivery outcomes [28–31]. Third, the inclusion of NPM-inspired strategies within decentralization reform suggests that a simple characterization of increased autonomy at the local level may not account for the multiple incentives and relational dynamics that characterize contemporary decentralization reforms [32,33]

Our research studies the case of health sector reform in Honduras to better understand the relationship between decentralization and decision autonomy among street-level bureaucrats (SLBs), namely the doctors, nurses, and social workers who provide primary healthcare services to the public. We utilize a quasi-experimental research design and apply Bossert’s [34] decision space approach to characterize decision autonomy for over 600 frontline health workers using original survey data in a matched sample of 65 decentralized and centrally-administered municipalities [35,36]. The Honduran case is emblematic of contemporary reform trends in Latin America: municipal governments, associations of municipalities, and NGOs can all serve as lead intermediary organizations under a common decentralization model for the management of local health centers and their staff, with the national ministry of health retaining regulatory authority and implementing performance-based incentives. This setting provides us unique and important leverage to credibly analyze decision autonomy reported by frontline staff and contribute to current debates about the nature and effectiveness of contemporary decentralization reforms in the Global South, as well as the drivers of decision autonomy by SLBs experiencing administrative reforms.

In summary, our work shows that reports of decision autonomy by frontline staff across four major functional areas of health service delivery – strategic and operational planning, organization and programming of services, human resources, and finances and budgeting – are slightly attenuated under decentralization. While lower relative to the centralized setting, decision autonomy within decentralized health facilities is generally reported to be in the moderate range and is not absent in any of the four functional areas. Importantly, this reduced autonomy is most pronounced when decentralization is led by a single municipal government, while systems led by NGOs show least difference from centralized systems in terms of frontline decision autonomy. One major implication of this research is that in order to understand the nature and consequences of contemporary versions of administrative reform, it is critical to consider the re-distribution of decision autonomy across actors at different levels within a hierarchical service delivery system.

## Autonomy, health service delivery, and decentralization in Honduras

Understanding the balance between central control and local autonomy, as well as the drivers of discretionary behavior on the part of civil servants and bureaucrats, has been a recurrent focus of research in public administration [e.g.,  26,27,37–40]. This is in large part because, across diverse settings around the world, the basic functioning of the administrative state rests on elected leaders and policymakers delegating some degree of authority to bureaucrats. Whether these implementing agents are doctors and nurses deciding about the priority of field-based clinics versus home visits, or caseworkers facilitating access to a social support program on behalf of their clients, they all exercise discretion and professional judgement when taking decisions about how to deliver public services and carry out the mandates of their organizations. In short, they have decision autonomy at the frontline of service delivery, and as Fukuyama [41] wrote recently in defense of the administrative state within the US context, “…bureaucratic autonomy is extremely important if the government is to function properly” (p. 2).

Research on decentralization, while voluminous, has only recently started to engage with perspectives from work on policy implementation and street-level bureaucracy [28–31,42]. This is an important linkage because many of the accountability- and information-enhancing arguments for this class of reforms hinge on the exercise of decision autonomy by frontline civil servants and bureaucrats [e.g., 2,6]. As such, moving research toward the facility and frontline levels to develop a better understanding of variation in how autonomy is experienced and enacted by SLBs is necessary to further knowledge of decentralization, particularly given how often findings on the service delivery effects of decentralization have been characterized as “mixed”  [43-45]. Finally, while trade-offs between different sources of control and autonomy have been central to debates about decentralization in the Global South, this is yet more important in light of contemporary versions of these reforms that create hybrid service delivery systems.

Our study focuses on the case of health sector decentralization reform in the Central American country of Honduras, which has the aim of improving healthcare by shifting authorities, responsibilities, and decision autonomy away from the national level and toward more local actors [46]. Since about 2007, the Honduran Ministry of Health (MOH) has been implementing a decentralization policy with the goals of strengthening primary healthcare service delivery, predominantly in rural, outlying, and underserved localities of the country [46,47]. In 2015 just ahead of data collection for the current study, the Honduran MOH had decentralized the administration and management of health centers in about 94 of the country’s 298 municipalities [36].

**Fig 1:**
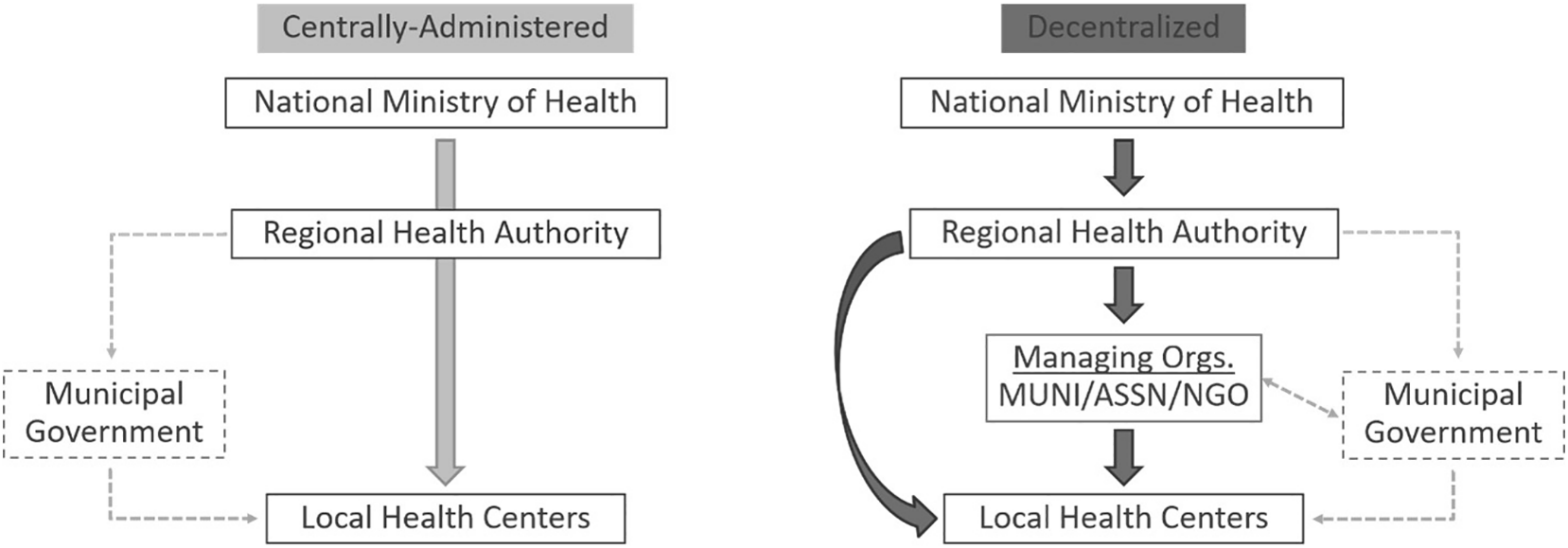
Centrally-administered and Decentralized Health Service Delivery in Honduras.

As described in prior research on this case and displayed in Fig 1, which is adapted from Molina-Garzón et al. [29], the health sector decentralization reform in Honduras involves two main changes: (1) the delegation of operational, planning, and supervisory functions for specific health centers from Regional Health Authorities to decentralized managing organizations via performance-based agreements, and (2) the deconcentration of oversight from the national-level MOH to the Regional Health Authorities over those same managing organizations and their health centers [46,48]. While the decentralization reform re-shaped work responsibilities for policymakers, administrators, and public managers, its immediate consequences were felt among frontline staff in primary care health centers. This is because public funds are transferred from the central MOH to decentralized managing organizations that are then responsible for the day-to-day work of the multiple health centers in their municipalities.

Specifically, decentralized managing organizations are tasked with supporting the facility-level staff on planning and organizing service delivery, ordering medications and supplies, identifying new staff to hire, monitoring and evaluation of all staff, overseeing finances and budgeting, and developing strategic partnerships or other sources of revenue. Given how rural these health centers are and the direct knowledge their staff has of the local population, frontline health workers can be and are involved in nearly all aspects of running the health center, including planning, taking decisions about how to organize service delivery, informing decisions about staffing, and managing the health center’s financial and material resources. The relevant Regional Health Authority retains a primary regulatory role over the decentralized managing organizations and also continues to oversee the health centers in general. In centrally-administered municipalities, the functions required to run the local health systems remain the same and are implemented by the staff of the Regional Health Authorities in concert with staff in local health centers.

Importantly, the MOH includes the possibility of three different types of organizations to serve as these decentralized managing organizations, or local intermediary organizations. In the Honduran context, single-municipal governments (MUNI), associations of mayors (ASSN), and non-governmental organizations (NGO) all can and do operate as decentralized managing organizations within a single, common decentralization policy. A shared template is used for the decentralization agreement across all settings, while the organizational form of the decentralized managing organization varies as part of the policy implementation process that occurs at the municipal and regional levels. For a given municipality identified for decentralization by the MOH based predominantly on need, the type of lead intermediary or managing organization results from a subsequent socialization and consultation process during implementation of the reform. Given this reality of the decentralization policy and its implementation, it is appropriate to consider decentralization as the main policy intervention in this case and the organizational form of the lead intermediary as a moderating condition. Additional details of the reform are available in [36]. In short, the Honduran case combines aspects of traditional political and administrative decentralization with characteristics inspired by the New Public Management, as has become common across Latin America [8,9,49].

## Understanding frontline decision autonomy under contemporary decentralization reform

Drawing from across the literatures on decision space in health policy and street-level discretion in public administration, there are four major factors likely shaping variation in decision autonomy among staff working within health systems: administrative form, namely decentralization, as well as capacity, resources, and accountability.

In terms of administrative form, several studies in the decision space literature have identified the classic policy-to-implementation gap whereby *de jure* decentralization does not translate directly to increased *de facto* decision autonomy at the local level [21,50,51]. Several factors suggest that this dynamic will be especially true in the context of contemporary decentralization reforms in Latin America. First, decentralization reforms increasingly include NPM-inspired strategies, like contracting-out and performance-based incentives, which emphasize control alongside autonomy [52,53]. Second, countervailing trends of re-centralization have been identified, whereby central actors attempt to assert their influence following waves of political or administrative decentralization [54,55]. These last two points align with the recognition in public administration that discretion can be utilized in both positive and negative ways by SLBs, something which has resulted in calls for greater control and accountability to support social equity in service delivery [56,57]. Third, there is a more general recognition that important governance and decision-making roles remain for central actors even in decentralized health systems [24,58]. Fourth, relative to the policymakers and administrators who have been emphasized in the decision space literature, frontline health workers are likely to privilege autonomy of action as much or more than autonomy of will [59]. These individuals routinely exercise discretion in the day-to-activities of translating policy mandates into services and responses for individual patients, and may be more likely to perceive changes in their institutional or organizational environments as limiting autonomy, at least in the short-to-medium term [26,27]. In summary, these factors jointly indicate that we should not be surprised to see **(H1)** *an attenuation of decision autonomy among frontline staff experiencing contemporary health sector decentralization reforms*.

Turning to capacity and resources, several articles in the decision space and bureaucratic discretion literatures have noted the importance of both factors for the realization of decision autonomy [20,22,60–62]. Whether this is technical expertise, past experience, or perceived decision-making authority, those individuals possessing relevant capacities are generally able to exercise greater decision autonomy than their counterparts in terms of health system governance and service delivery. Closely related, though nonetheless distinct, is the topic of resources. These same articles have generally confirmed that the presence of adequate financial or material resources is a necessary precondition to individuals’ abilities to act on whatever *dejure* autonomy they may have in different functional areas. As such, we similarly expect that both **(H2)** *capacity and* **(H3)** *resources will have a positive association with decision autonomy among frontline health workers*.

Lastly, accountability has been suggested as potentially restricting [22] or supporting [62] the exercise of decision autonomy in different contexts. We do not view this as a contradiction necessarily, but rather a reflection of the multiples roles that accountability can play in the governance of public service delivery [31,56,63–65]. Among frontline staff, accountability is most commonly enacted through monitoring [30,66], whereby managers, political appointees, or members of the public observe and comment on the actions of health workers. This monitoring can be experienced in at least two different ways: as enforcement or support. The enforcement experience of monitoring aligns with the prototypical, formal evaluation visit where those with authority over health staff “check-up” on activities in the health center, utilize monitoring forms that often include actual check boxes, and debrief with staff about what they have done well and where there is room for improvement. In the supportive experience of monitoring, a site visit or consultation by superiors with health staff takes a different tone, typically one focused on the development of mutual understanding and with the goal of joint problem-solving. In the extreme, the former experience can be felt as constraining and potentially even punitive, whereas the latter experience has the potential to be empowering. Given this, we expect that **(H4)** *accountability which is experienced as evaluation or enforcement will serve to constrain decision autonomy, whereas supportive accountability oriented toward problem-solving will be associated with expanded decision autonomy among frontline health workers*.

## Empirical strategy

Health sector decentralization reform in Honduras is a strong case for assessing the relationship between decentralization and frontline decision autonomy in health service delivery. First, by focusing on a single country we minimize the influence of potential unobserved confounding variables related to reform targeting, background conditions, and historical factors [2,67]. Second, the partial implementation of the reform itself allows for a quasi-experimental design in the identification of a credible comparison group of centrally-administered municipalities against which to assess the experience of decentralized municipalities, something that has been rare in the broader research on health sector decentralization [35,36]. And third, this study serves to add an important case to the growing Latin American public administration literature on governance reform and its implications for social service delivery.

### Sample of municipalities and health workers

This study uses data collected from an original survey conducted with a sample of health workers in 65 of 298 municipalities across Honduras. Forty-two sampled municipalities were decentralized and 23 comprised a matched group of centrally-administered municipalities. The selection of this matched sample of 65 municipalities was conducted by researchers prior to the present study’s analysis utilizing propensity score matching in a quasi-experimental research design [35,36]. Specifically, those researchers conducted interviews with policymakers from the Honduran health sector to understand the drivers of targeting and rollout for the health sector decentralization reform in question. Drawing on those interviews and existing quantitative data, they conducted a propensity score matching analysis to identify a well-balanced set of municipalities for additional, original data collection. In terms of the decentralized sample of municipalities, researchers included all municipalities that had been under the reform for at least four years at the time of data collection in 2016−17. Then, logistic regression was used to model the decentralization targeting decision at the municipal level based on all available covariates, and the propensity scores from that final model supported the matching analysis that produced the final weights that are applied to all models presented in this paper. As described in prior work drawing on this same data, “…the resulting sample is well-balanced on major pre-treatment characteristics: health facilities, health services, demographic attributes, distances to major population centers, electoral variables, civil society organizations, cash transfer beneficiaries, social-economic indicators, municipal capacity indicators in terms of human development and own-source revenue, and exposure to two related health interventions” [68, p. 6].

Across the 65 municipalities in this sample that covers 14 of 18 states in the country, Honduran survey staff conducted an original survey with 627 health workers from more than 300 health centers in 2016–17. This included almost all public health facilities providing primary care services in these localities, and a systematic sample of the doctors, nurses, social workers, and other staff working in those health centers (about 2 staff members per health center). As discussed earlier, these health centers are predominantly rural and have relatively few staff members: typically one or even no doctors, 1–3 nurses, and perhaps one community health worker (social worker). As such, there was relatively little decision-making on the part of the survey staff about identifying potential respondents within the health centers, even though lists of all staff were not available from the MOH. Given this, and as has been described in prior work utilizing this data, “…survey staff took all precautions possible to ensure a systematic and unbiased sample of health workers…” when carrying out their field-based data collection activities [68, p. 6]. This study was approved by the University of Colorado Boulder Institutional Review Board (IRB) with local review conducted through the Honduran Ministry of Health Undersecretary for Integrated Health Service Networks. It was determined that verbal consent was appropriate in this context.

When disaggregating by the different types of lead intermediary organizations under decentralization, about 32-percent of the sampled health workers represent centrally-administered health centers, about 20-percent come from decentralized health centers led by a single municipal government, approximately 28-percent were from decentralized health centers led by an association of mayors, and the remaining 20-percent of the sample of health workers came from decentralized health centers led an NGO. Table 1 presents descriptive statistics for the sample of health workers.

**Table 1:** Descriptive Statistics from a Sample of Health Workers across 65 Centrally-administered and Decentralized Municipalities in Honduras.

Variable	N	Mean	Median	SD	Min	Max
*Respondent Characteristics*						
Age	596	34.30	32	9.62	16	73
Female (0–1)	624	0.74	1	0.43	0	1
Education (1–5)	623	3.78	4	0.77	1	5
Role – Doctor (0–1)	627	0.10	0	0.30	0	1
Role – Nurse (0–1)	627	0.57	1	0.50	0	1
Role – Social Worker (0–1)	627	0.21	0	0.41	0	1
Role – Other Staff Type (0–1)	627	0.12	0	0.32	0	1
*Measures of Autonomy*						
Perceived Decentralization (1–5)	554	3.58	4	0.77	1	5
Decision Autonomy – Planning (1–3)	618	2.09	2	0.85	1	3
Decision Autonomy – Finances (1–3)	610	1.47	1	0.91	1	3
Decision Autonomy – Human Resources (1–3)	618	1.67	1	0.87	1	3
Decision Autonomy – Organizing Services (1–3)	620	2.34	2	1.12	1	3
*Measures of Capacity*						
Years in Health Sector	627	8.12	5	7.69	0	37
Perceived HC Staff Leadership (1–3)	627	4.13	4	0.66	2	5
*Measures of Resources*						
Perceived HC Resources (1–5)	627	2.78	3	0.72	1	5
Perceived Freq. Use HC Services (1–5)	627	3.34	3	0.68	2	5
*Measures of Accountability*						
Total Evaluation Visits	627	9.76	10	7.55	0	84
Total Support Visits	627	7.45	5	8.20	0	84

Notes: Ranges for categorical variables listed in parentheses next to the variable name.

### Measuring autonomy

Our work follows Maggetti & Verhoest [69]  in defining autonomy as the ability to “…translate ones’ own preferences into authoritative actions, without external constraints” (p. 239). External constraints – be they formal regulations, informal rules and norms, or administrative reforms – can serve to curtail or place boundaries on the autonomy with which frontline line workers engage in their day-to-day work. In contrasting sociological and principal-agent perspectives on autonomy, Ege [59] emphasizes the distinction between “…the capacity to develop autonomous preferences (autonomy of will) and the ability to translate these preferences into action (autonomy of action)” (p. 557). Our conceptual emphasis here is predominantly on autonomy of action under decentralization reform, namely frontline decision autonomy. For operationalizing this concept, we utilize Bossert’s [34] decision space approach that “…focuses on the range of choice that is available to local decision-makers along a series of functional dimensions,” and helps identify the “…range of discretion or choice allowed to agents in the process of decentralization which differentiates decentralized…relationships from centralized relationships…” (p. 1514, 1517−18).

Building on the work of Bossert and others who have sought to credibly measure autonomy using the decision space approach, [35,36] developed survey questions to assess the range of decision autonomy on four functional areas relevant to health service delivery at the local health center level: operational and strategic planning, organization and programming of services, human resources, and finances and budgets. These were selected to reflect both the range of major task areas where frontline staff could plausibly exercise decision autonomy given the study context of rural Honduras, and were informed by the extant literature applying the decision space approach across different cases of health sector decentralization. For each functional area, health workers were presented with a survey question including short descriptions reflecting low, medium, and high decision autonomy and then asked to report the option that best reflected their experiences. Draft versions of the short descriptions that characterized the 3-point scale for each functional area were initially developed by researchers, discussed in focus groups with health personnel in Honduras, revised, and pilot tested before being finalized for the survey.

To take one example, decision autonomy in terms of strategic and operational planning was characterized in reference to the health center’s annual operating plan. This is a document crafted by the local health center staff each year in centrally-administered and decentralized settings. On the survey question, the low decision autonomy option was described as the health center’s annual operating plan not being developed in the health center, but rather being sent to the health center from above by the closest managing organization and then only executed in the health center. In contrast, the high decision autonomy option for this functional area was described as the annual operating plan being developed principally by the health center staff themselves and based on or reflecting in its majority the conditions of the local population. Researchers developed similar scales for the survey questions corresponding to the other three functional areas, in each case characterizing what low, medium, and high decision autonomy would look like for an emblematic task in that functional area, and asking respondents to select which of the options best reflected their experience in the health center. Additionally, health workers were asked to characterize the degree of decentralization of their local health centers on a five-point scale as a complementary indicator of the administrative context in which they were taking decisions. The original survey questions are included in the supporting information.

### Measures of local health system administration

The key independent variable for this analysis is the administrative form by which the municipal health system is managed. We approach this in two ways. First, we use a dichotomous measure of whether the municipal health system, and its health centers, is decentralized; the municipality is coded “0” if it remains centrally-administered and “1” if decentralized. Second, we create a categorical variable to identify the distinct types of intermediary managing organizations. This variable includes whether the municipality is centralized (CENT) or managed under decentralization by a single municipal government (Decent-MUNI), an association of mayors (Decent-ASSN), or a non-governmental organization (Decent-NGO).

### Measures of capacity, resources, and accountability

We also consider the three other major factors expected to be associated with decision autonomy: capacity, resources, and accountability. Given our focus on frontline staff and informed by recent studies of decision space [20,60,62], we utilize two indicator variables for each of these three concepts. In terms of capacity, we include the number of years an individual has spent working within the health sector and the degree of leadership they perceive the staff of the health center to have on a five-point scale. For resources, we asked respondents to report the amount of resources they perceive the health center to have relative to its responsibilities and the degree of health service utilization, or demand, on the part of the public, both on five-point scales. Lastly, we include respondents’ reports of the number of evaluative and supportive monitoring visits they experienced during the last year.

### Control variables

We include four control variables to account for demographic differences between respondents. Specifically, we control for age, female, degree of education, and role type of each respondent. Age is a continuous variable measured in years. Female is a dichotomous variable where the data is coded as a 1 if the respondent identifies as a female and 0 otherwise. Education is an ordinal variable: 1 “no education”, 2 “primary education, 3 “secondary education”, 4 “technical degree”, and 5 “university-level education.” For sensitivity analyses, we replace these demographic control variables with role type controls, namely whether the respondent was a doctor, nurse, social worker, or other type of position.

## Results

In this section, we present the results of our analysis examining the relationship between decentralization and decision autonomy among frontline health workers. We utilize standard regression methods to analyze decision autonomy based on data from over 600 doctors, nurses, social workers, and other staff across a matched set of 65 municipalities in Honduras. All regression models include weights based on the propensity score matching analysis that provide for the greatest possible balance between decentralized and centrally-administered settings, as well as standard errors clustered at the municipality level.

### Decentralization is associated with lower decision autonomy

Our first analysis considers the direct relationship between decentralization reform and decision autonomy among the frontline staff. Models 2–5 in Table 2 show a negative association between decentralization and decision autonomy. These reductions are significant at conventional levels for strategic planning and organizing service delivery, but less so for finances and human resources. As model 1 in Table 2 shows, health workers in decentralized settings did indeed report that their health systems experienced a greater degree of decentralization relative to those in centralized settings, even though the reform was associated with lower reported decision autonomy. This initial analysis is consistent with the expectation developed for H1 that decision space can narrow under decentralization reform. Furthermore, it provides a baseline understanding of the relationship between decentralization reform and decision autonomy, and leads to the subsequent analyses where we focus on variation in intermediary organization types under decentralization.

**Table 2:** The Relationship between Decentralization Reform and Decision Autonomy Reported by Frontline Health Workers.

*Decision Autonomy*	(1) Decentralization Perception	(2) Autonomy Planning	(3) Autonomy Organizing Services	(4) Autonomy Human Resources	(5) Autonomy Finances
Decentralized (ref. cent.)	0.91^***^	−0.42^**^	−0.29^**^	−0.29	−0.14
	(0.26)	(0.14)	(0.09)	(0.20)	(0.10)
Female	−0.09	−0.10	−0.14	−0.08	−0.23^*^
	(0.15)	(0.06)	(0.09)	(0.10)	(0.09)
Education	0.16	0.10^^^	0.04	0.24^**^	0.05
	(0.13)	(0.06)	(0.05)	(0.08)	(0.05)
Age	−0.00	−0.01	−0.00	−0.01	−0.00
	(0.01)	(0.01)	(0.01)	(0.01)	(0.01)
Years in Health Sector	−0.00	0.01	0.01	0.02^*^	0.01
	(0.02)	(0.01)	(0.01)	(0.01)	(0.01)
Nurse (ref. doctor)	0.17	−0.04	0.01	0.04	0.22^^^
	(0.24)	(0.17)	(0.11)	(0.20)	(0.12)
Social Worker (ref. doctor)	0.08	−0.07	−0.13	0.05	−0.08
	(0.20)	(0.17)	(0.14)	(0.17)	(0.14)
Other Staff Type (ref. doctor)	0.55	−0.16	−0.29^*^	0.38	0.35^^^
	(0.42)	(0.19)	(0.14)	(0.23)	(0.20)
Constant	2.37^***^	2.30^***^	2.60^***^	1.03^^^	1.39^***^
	(0.47)	(0.43)	(0.37)	(0.57)	(0.32)
*N*	526	581	584	582	574
*R* ^2^	0.15	0.09	0.08	0.12	0.07

Notes: Weighted OLS regression with clustered standard errors by municipality in parentheses; ^p < 0.10, *p < 0.05, **p < 0.01, ***p < 0.001; “ref.” is the abbreviation that indicates the excluded reference category for the variable, “cent.” refers to centralized administration

### Decentralization led by single municipal governments is associated with greater reductions in decision autonomy relative to other varieties of decentralization

Table 3 presents the results of our second regression analysis where we disaggregate the administrative form indicator into four types: centralized administration (excluded base category), municipal government-led, association-led, and NGO-led decentralization. The results show that health workers under municipal government-led decentralization report lower decision autonomy in each of the four functional areas than health workers experiencing centralized administration, those in association-led systems report lower decision autonomy in terms of planning and finances relative to centralized counterparts, and health workers in NGO-led systems report the fewest differences in their decision autonomy compared to centrally-administered settings. As in the preceding analysis, it is important to note that health workers in all three types of decentralization do indeed report greater levels of decentralization relative to those in centrally-administered settings. Overall, these findings confirm that among decentralized settings, those led by single municipal governments are likely to feel some degree of constraint in their decision autonomy with respect to major health center functions, relative to health workers in centrally-administered settings.

**Table 3:** The Relationship between Decentralization by Organization Type and Decision Autonomy Reported by Frontline Health Workers.

*Decision Autonomy*	(1) Decentralization Perception	(2) Autonomy Planning	(3) Autonomy Organizing Services	(4) Autonomy Human Resources	(5) Autonomy Finances
Decent-MUNI (ref. cent.)	0.80^*^	−0.41^*^	−0.61^***^	−0.58^**^	−0.29^*^
	(0.31)	(0.18)	(0.18)	(0.21)	(0.12)
Decent-ASSN (ref. cent.)	1.07^***^	−0.42^**^	−0.15	−0.17	−0.28^*^
	(0.27)	(0.16)	(0.10)	(0.22)	(0.11)
Decent-NGO (ref. cent.)	0.72^*^	−0.41^^^	−0.32^*^	−0.28	0.21
	(0.28)	(0.21)	(0.13)	(0.23)	(0.13)
Female	−0.06	−0.10	−0.11	−0.05	−0.25^**^
	(0.15)	(0.10)	(0.09)	(0.10)	(0.09)
Education	0.16	0.10^^^	0.05	0.25^**^	0.07
	(0.13)	(0.06)	(0.05)	(0.08)	(0.05)
Age	0.00	−0.01	0.00	−0.01	−0.00
	(0.01)	(0.01)	(0.01)	(0.01)	(0.01)
Years in Health Sector	−0.00	0.01	0.01	0.02^*^	0.01
	(0.02)	(0.01)	(0.01)	(0.01)	(0.01)
Nurse (ref. doctor)	0.20	−0.04	0.06	0.10	0.22^*^
	(0.25)	(0.17)	(0.11)	(0.20)	(0.11)
Social Worker (ref. doctor)	0.15	−0.07	−0.03	0.13	−0.10
	(0.21)	(0.16)	(0.14)	(0.16)	(0.14)
Other Staff Type (ref. doctor)	0.61	−0.16	−0.22	0.43^^^	0.30
	(0.42)	(0.18)	(0.14)	(0.23)	(0.20)
Constant	2.24^***^	2.31^***^	2.45^***^	0.90	1.46^***^
	(0.64)	(0.42)	(0.38)	(0.57)	(0.29)
*N*	526	581	584	582	574
*R* ^2^	0.17	0.09	0.12	0.14	0.14

Notes: Weighted OLS regression with clustered standard errors by municipality in parentheses; ^p < 0.10, *p < 0.05, **p < 0.01, ***p < 0.001; “ref.” is the abbreviation that indicates the excluded reference category for the variable, “cent.” refers to centralized administration

**Fig 2:**
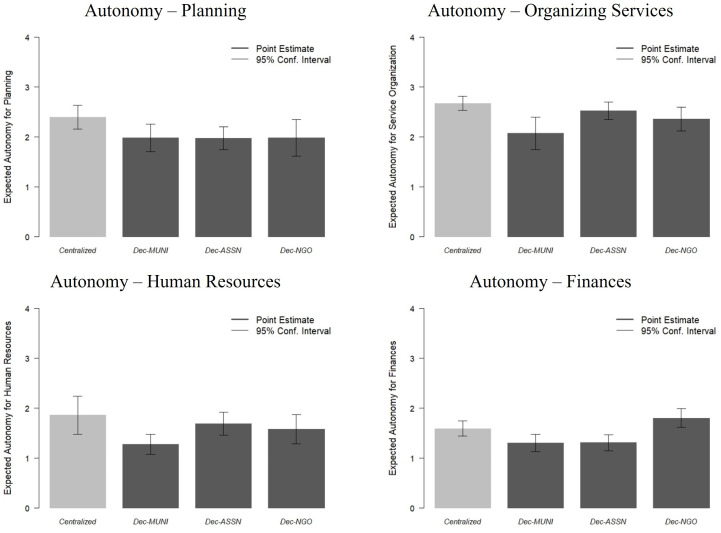
Decision Autonomy by Administrative Form.

Fig 2 displays the substantive relationships associated with the results from Table 3. Using a quantity-of-interest simulation approach, this figure compares the reported degree of autonomy that is predicted for a typical respondent in the sample between a centrally-administered setting and each of the three decentralized settings, including 95-percent confidence intervals [70]. First, this figure clearly shows that decision autonomy is lower overall for finances and human resources, both below two on the three-point scale, while decision autonomy is higher across the board for planning and organizing service delivery where values are generally two or above. Second, across the functional areas, decentralization led by municipal governments is associated with a reduction of about 0.40 to 0.60 in predicted decision autonomy compared to centrally-administered settings, which corresponds to between 12- and 20-percent on the questions’ three-point scales. Third, NGO-led decentralization is the administrative form where the level of decision autonomy is most similar to that reported by health workers in centrally-administered health centers.

### Doctors report the greatest reductions in decision autonomy under decentralization, though there are relatively few differences by staff role type

**Fig 3:**
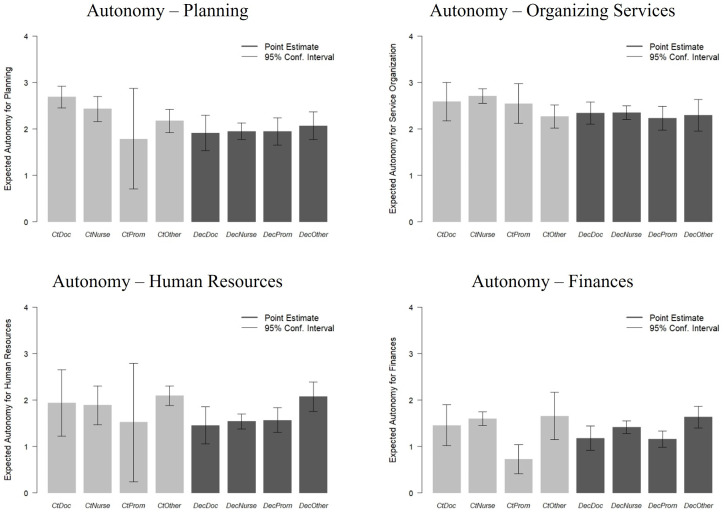
Decentralization and Reported Decision Autonomy by Staff Role Type.

Table 4 presents the next component of the analysis where we assess differences in the decentralization-to-decision autonomy relationship by the major staff role types: doctors, nurses, social workers, and others. Fig 3 shows the expected level of autonomy for each staff type between decentralized and centrally-administered settings based on this analysis. Looking across the four functional areas, the evidence generally suggests that reported decision autonomy is more even across staff types under decentralization, while there are greater differences under centralized administration with doctors reporting higher levels, then nurses, and followed by social workers and others. Additionally, considering strategic and operational planning, there are greater reductions in decision autonomy among doctors between decentralized and centrally-administered health centers as compared with the other staff types. However, the general message from this analysis is that there are relatively modest differences in decision autonomy across staff types.

**Table 4:** The Relationship between Decentralization Reform and Decision Autonomy among Role Types Reported by Frontline Health Workers.

*Decision Autonomy*	(1) Decentralization Perception	(2) Autonomy Planning	(3) Autonomy Organizing Services	(4) Autonomy Human Resources	(5) Autonomy Finances
Decentralized (ref. cent.)	1.37^***^	−0.78^***^	−0.24	−0.48	−0.28
	(0.33)	(0.20)	(0.22)	(0.39)	(0.26)
Nurse (ref. doctor)	0.50	−0.26	0.12	−0.05	0.14
	(0.49)	(0.22)	(0.26)	(0.40)	(0.25)
Social Worker (ref. doctor)	−0.21	−0.90	−0.04	−0.42	−0.73^*^
	(0.56)	(0.56)	(0.29)	(0.72)	(0.31)
Other Staff Type (ref. doctor)	1.09^^^	−0.52^*^	−0.32	−0.16	0.20
	(0.66)	(0.21)	(0.26)	(0.36)	(0.38)
Decent. X Nurse	−0.46	0.29	−0.12	0.14	0.09
	(0.46)	(0.28)	(0.27)	(0.46)	(0.27)
Decent. X Social Worker	0.21	0.94	−0.07	0.53	0.71^*^
	(0.56)	(0.58)	(0.29)	(0.74)	(0.34)
Decent. X Other Staff Type	−0.96	0.67^*^	0.27	0.46	0.25
	(0.61)	(0.29)	(0.29)	(0.43)	(0.41)
Female	−0.05	−0.14	−0.15	−0.11	−0.25^**^
	(0.16)	(0.10)	(0.09)	(0.10)	(0.09)
Education	0.14	0.12^^^	0.05	0.25^***^	0.06
	(0.12)	(0.06)	(0.05)	(0.07)	(0.05)
Age	−0.00	−0.01	0.00	−0.01	−0.00
	(0.01)	(0.01)	(0.01)	(0.01)	(0.01)
Years in Health Sector	0.00	0.01	0.01	0.02^*^	0.01
	(0.02)	(0.01)	(0.01)	(0.01)	(0.01)
Constant	2.13^**^	2.52^***^	2.46^***^	1.11^*^	1.49^***^
	(0.78)	(0.42)	(0.49)	(0.55)	(0.38)
*N*	526	581	584	582	574
*R* ^2^	0.16	0.10	0.09	0.12	0.08

Notes: Weighted OLS regression, binary indicator of decentralization, role type controls models with clustered standard errors by municipality in parentheses; ^p < 0.10, *p < 0.05, **p < 0.01, ***p < 0.001; “ref.” is the abbreviation that indicates the excluded reference category for the variable, “cent.” refers to centralized administration.

### Important associations exist between capacity, resources, and accountability in supporting expanded decision autonomy in certain cases

Lastly, Table 5 presents the analysis of decision autonomy across the four functional areas adding in measures of capacity, resources, and accountability. Based on this, the main results of slightly attenuated decision autonomy under decentralization, concentrated particularly in systems led by municipal governments, are confirmed. In terms of capacity, prior experience working in the health sector is unrelated to decision autonomy in any functional area, while health center staff leadership is positively associated with decision autonomy in the two functional areas most proximate to the facility level, strategic and operational planning and programming and organizing health service delivery. This is in line with the expectations developed in H2, if not entirely confirmatory given the null result for prior experience.

**Table 5:** The Relationship between Decentralization Reform, Capacity, Resources, and Accountability and Decision Autonomy Reported by Frontline Health Workers.

*Decision Autonomy*	(1) Autonomy Planning	(2) Autonomy Organizing Services	(3) Autonomy Human Resources	(4) Autonomy Finances
*Administrative Form*				
Dec-MUNI (ref. cent.)	−0.47^**^	−0.75^***^	−0.63^***^	−0.30^*^
	(0.17)	(0.17)	(0.18)	(0.14)
Dec-ASSN (ref. cent.)	−0.43^**^	−0.26^*^	−0.27	−0.29^*^
	(0.15)	(0.12)	(0.18)	(0.12)
Dec-NGO (ref. cent.)	−0.47^*^	−0.46^**^	−0.42^*^	0.16
	(0.20)	(0.14)	(0.20)	(0.13)
*Capacity*				
Years in Health Sector	0.01	0.00	0.02^^^	0.01
	(0.01)	(0.01)	(0.01)	(0.01)
Staff Leadership	0.19^*^	0.16^*^	0.05	0.07
	(0.09)	(0.07)	(0.06)	(0.07)
*Resources*				
Health Center Resources	0.17^**^	0.11^*^	0.28^***^	−0.00
	(0.05)	(0.05)	(0.05)	(0.04)
Frequency Health Center Use	−0.04	−0.01	−0.10	0.13
	(0.07)	(0.05)	(0.06)	(0.09)
*Accountability*				
Total Evaluation Visits	−0.00	0.01	−0.01	−0.01
	(0.01)	(0.01)	(0.01)	(0.01)
Total Support Visits	0.01	0.01	0.02^^^	0.01^*^
	(0.01)	(0.01)	(0.01)	(0.01)
*Demographic Controls*				
Age	−0.00	0.00	−0.00	−0.00
	(0.01)	(0.01)	(0.01)	(0.01)
Female	−0.07	−0.06	−0.02	−0.27^**^
	(0.09)	(0.09)	(0.09)	(0.09)
Education	0.03	0.02	0.16^^^	0.03
	(0.07)	(0.05)	(0.09)	(0.04)
*Role Type Controls*				
Nurse (ref. doctor)	−0.12	0.08	0.02	0.24^*^
	(0.15)	(0.11)	(0.16)	(0.10)
Soc. Worker (ref. doctor)	−0.18	−0.03	0.05	−0.11
	(0.16)	(0.14)	(0.15)	(0.14)
Other Staff (ref. doctor)	−0.24	−0.20	0.31	0.25
	(0.17)	(0.14)	(0.24)	(0.16)
Constant	1.40^**^	1.52^***^	0.56	0.84^^^
	(0.49)	(0.40)	(0.50)	(0.46)
*N*	574	577	575	567
*R* ^2^	0.17	0.19	0.25	0.17

Notes: Weighted OLS regression with clustered standard errors by municipality in parentheses; ^p < 0.10, *p < 0.05, **p < 0.01, ***p < 0.001; “ref.” is the abbreviation that indicates the excluded reference category for the variable, “cent.” refers to centralized administration

Turning to the question of resources, staff reporting their facilities had greater levels of material resources relative to their responsibilities also indicate higher levels of decision autonomy across three of the four functional areas. The case where this association is not significant is for finances and budgeting, which contributes to the view of that functional area being distinct from the others. Staff reports of the level of utilization or demand for services was unrelated to decision autonomy. As with capacity, the results on resources are in line though not entirely confirming of H3. Finally, our analysis shows the dual roles of accountability: evaluative monitoring visits are negatively, though not significantly, associated with decision autonomy, while supportive monitoring visits are positively associated with decision autonomy in terms of human resources and finances and budgeting. These results are similarly in line but not entirely confirming of H4.

### Limitations

These findings of this research should be interpreted in light of certain limitations. First, while we rely on a novel and credible source of data about decision autonomy across different functional areas, the information analyzed here nonetheless remains based on self-reports of autonomy rather than observed behavior. Second, while this analysis is grounded in a broader, quasi-experimental research design, something relatively rare in studies of decentralization, we do rely on data for a single time point. Future research on the relationship between decentralization and decision autonomy would benefit from examining trends over time, utilizing stronger strategies for causal inference where possible, and incorporating qualitative data such as interviews with frontline health workers to more fully characterize the factors underlying reductions in reported decision autonomy. Third, our analysis of the conditional association between decision autonomy on the one hand, and administrative form, capacity, resources, and accountability on the other, remains cross-sectional and does not directly model the inter-relationships between these factors. Future research can consider the potential utility of structural equation modeling (SEM) in this regard, as in recent work by Feldhaus et al. [60]. Fourth, our research focuses on well-established functional areas in decentralized health service delivery and develops decision space questions for each that are sensitive to the study context and informed by interviews and focus groups with frontline health workers. That said, future work would benefit from considering additional functional areas relevant to frontline health workers’ jobs, including decisions about diagnosis and treatment plans for individual clients and patients. Finally, the findings presented here are drawn from an analysis of subnational variation in health sector reform in Honduras. This is a substantively important case given its organizational variation in decentralized health service delivery, and contributes an additional Latin American case to the public administration and health policy literatures. Nonetheless, future research is warranted to assess the arguments presented here across different national and regional contexts.

## Discussion

Our analysis shows that relative to centrally-administered municipalities, decentralization reform is credibly associated with slight decreases in health workers’ decision autonomy in four functional areas: strategic and operational planning, organization and programming of services, human resources, and finances and budgets. This is evidence in support of the paper’s first hypothesis. While the precise reasons for this reduction in reported decision autonomy remain an area of ongoing research, several plausible explanations do emerge. First, the decentralization reform in the Honduran case results in a hybrid service delivery model that combines performance-based incentives with deconcentration and delegation of service delivery functions. As such, it may be that the performance management features of the reform outweigh the delegation features when it comes to frontline decision autonomy, in line with prior work on the balancing of central control and local autonomy within public sector reform [e.g., 71,72]. Second, previous research on the Honduran case has shown credible evidence of increases in supportive and evaluation-oriented monitoring of health center staff by the decentralized managing organizations [30]. This greater frequency and salience of one type of accountability, even within a setting of delegated functions, could be associated with a reported reduction in decision autonomy among frontline staff, which is supported by theoretical work on the role of intermediary organizations in co-production arrangements [65]. Third, one of the major functions that is pushed down toward more local actors under the reform is the authority and decision-making about staffing, and prior research has shown that there is a change in the composition of staff within health centers associated with the reform. When given the authority, decentralized managing organizations appear to both attract and select staff that tend to be somewhat younger, have somewhat less experience, and they give greater priority to community health workers [28]. While this change in staff composition has been a mechanism plausibly related to key improvements under the reform, it may also come at the expense of some minor degree of decision autonomy as seen here.

At the same time that they report somewhat lower decision autonomy, our results also show that frontline health workers in decentralized settings do report that there is a greater degree of decentralization in the administration of their municipal health systems compared to counterparts within centrally-administered settings. When coupled with our main findings, this latter points serves as evidence validating an experience of somewhat diminished decision autonomy under decentralization, and evidence against an interpretation of this finding as stemming from a weakly implemented reform or a general lack of information on the part of frontline staff vis-à-vis policymakers in the capital city. Health workers in decentralized settings do report that their health centers are significantly more decentralized than their counterparts from centralized settings.

The minor attenuation of decision autonomy under decentralization is more pronounced in the functional areas of strategic and operational planning and organization and programming of services, relative to human resources and finances and budgets. Additionally, the reductions in decision autonomy identified here appear predominantly in decentralized systems led by municipal governments, and to a lesser degree those led by associations of municipalities; decentralized health systems led by NGOs report the least difference in decision autonomy compared to centrally-administered health systems. Prior research on the case of health sector decentralization in Honduras has shown improvements in health service delivery and key health outcomes among NGO-led systems, and also association-led systems for some indicators, relative to the centrally-administered comparison group [11,30]. Local health systems under municipal government-led decentralization have generally been shown as indistinguishable from the centrally-administered comparison group in these studies. Additionally, recent work also showed that reports of corruption were higher under municipal government-led decentralization in Honduras, and that civil society engagement played a role in attenuating reported corruption across decentralized settings [68]. Combined with the present study’s findings on decision autonomy, a reasonable question is whether risks of politicization, patronage, or outright corruption under decentralization [73–75] may help explain some of the reduced decision autonomy seen here within municipal government-led systems. Moreover, it will be important for future work to consider whether and how involving nonstate intermediary organizations within decentralization reforms may be able to ameliorate some of these negative dynamics, particularly given the prevalence of hybrid service delivery models resulting from contemporary administrative reforms across Latin America.

There is also limited evidence that the decreases in autonomy under decentralization are more pronounced for doctors relative to the other health staff roles, particularly in strategic and operational planning, though differences among staff types were modest and largely overshadowed by organizational and institutional factors. In principle, this is reasonable as doctors could be expected to exercise the greatest autonomy or discretion within a primary care health center based on the education and training that they have, and that the lead staff member within the health facility is most likely to be a doctor (if it is one of the minority of facilities that has a doctor among its staff). Given that, doctors would perhaps be most attuned to reductions in their autonomy as administrative reforms are implemented, particularly those that introduce new, intermediary actors into the service delivery systems as has been the case with other types of managers experiencing administrative reforms [71,72]. Then, in fact, it is even more noteworthy how limited the staff-level differences are in reported autonomy in this case, and that they are largely trumped by factors related to the reform and more general conditions of the work environment.

In addition to administrative reform, we show that material resources are a general precondition to expanded decision autonomy across most functional areas, which supports the paper’s second hypothesis, and that capacity in terms of staff leadership matters for those functional areas closest to the facility level, namely planning and organizing service delivery, which is generally in line with the third hypothesis while demonstrating differences across functional areas. Also, our work shows that supportive accountability can serve to increase decision autonomy on the part of frontline staff in those functional areas that are more likely influenced by managers or administrators above the facility level, specifically human resources and finances and budgets, a finding that is consistent with the paper’s fourth hypothesis, though again showing important differences across functional areas. This last point highlights the importance of management within decentralized health systems, and that the behavioral foundations of improved service delivery may be as much about supporting staff in their day-to-day work as it is about aligning incentives and strengthening oversight.

It is important to emphasize here that high levels of decision autonomy are not necessarily a positive in their own right [60,76], and there are numerous examples of street-level autonomy being used to engage in discretionary behaviors that are unfavorable to clients and work to undermine social equity [56,57,77]. Health systems are just that, systems, where the service delivery experienced by an individual patient or client is ultimately the product of choices and actions of multiple actors. For some civil servants, high levels of autonomy may be empowering, but for many others that can reflect circumstances of limited oversight, limited support, and few connections to others within the health system, namely, the kinds of circumstances that many decentralization reforms are aiming to remedy. In efforts to better understand situations like this one, as well as numerous others in public administration relating to the governance of local service delivery beyond public health, our work has aimed to highlight the decision space approach as a productive way to characterize and analyze decision autonomy within decentralized regimes. As we have shown here, the decision space approach can help support the analytical basis to address different types of context-sensitive tradeoffs related to balancing central control and local autonomy within and across levels of the service delivery hierarchy, and ultimately help identify which types of hybrid arrangements and what circumstances lead to more equitable service delivery for the public under decentralization.

Since its development in the late 1990s, the decision space approach [34] has been utilized extensively to operationalize autonomy and analyze health sector decentralization across multiple countries [78]. This approach goes beyond traditional conceptualizations of decentralization in emphasizing changes in the range of choice allocated to and realized by actors within administrative systems. In applying the decision space approach, range of choice is typically characterized for different functional areas as relevant to the case. Common functional areas appearing in the literature include planning, budgeting, human resources, service delivery, and information management, among others. Furthermore, the decision space approach highlights contextual conditions as important to the range of choice that can be realized, both in terms of generating incentives and potential constraints that can influence local autonomy [79]. The decision space approach has not, however, featured prominently within the public administration literature where studies of bureaucratic autonomy have tended to utilize aggregate indicators specific to their case contexts. As this work has led to important insights, we suggest that the decision space approach and its focus on functional areas can provide both the flexibility to apply across cases and policy areas and the degree of standardization that would support building more cohesive and comprehensive findings on decision autonomy for the types of decentralized governance systems commonly studied by scholars of public administration.

## Conclusion

Drawing on the case of health sector reform in the Central American country of Honduras, this study examines the relationship between decentralization and decision autonomy on the part of frontline health workers. We apply Bossert’s [34] decision space approach and utilize a quasi-experimental research design and original survey data from frontline health staff, street-level bureaucrats, to understand how specific reform features influence the *defacto* decision autonomy reported by doctors, nurses, and social workers charged with delivering primary care health services to patients in local health centers [54,73–75].

Our results show that decentralization is credibly associated with a modest attenuation of decision autonomy for frontline health workers across four primary functional areas: strategic and operational planning, organization and programming of health services, human resources, and finances and budgeting. Even if slightly attenuated relative to centralized administration, decision autonomy remains in the moderate range under decentralization for most of the functional areas examined. Furthermore, the reduction in decision autonomy is most pronounced in decentralized systems led by single municipal governments, while systems led by associations and particularly by NGOs show fewer differences in decision autonomy as compared to centrally-administered settings. When considering different staff role types, we see slight evidence for doctors having higher decision autonomy than nurses or social workers, and then experiencing greater corresponding reductions under decentralization, though these differences are minor relative to system- and organization-level factors overall. It is important that future research prioritize further disentangling the relationship among administrative form, capacity, resources, and accountability, as well as understanding how decision autonomy related to multiple dimensions of health system performance.

The findings of this research are important for both the academic literatures on decision space and autonomy in public administration, as well as the policy and practice of health sector decentralization in the Global South. Our work shows that frontline staff, who are critical actors at the end of the service delivery chain, should be more central in studies of health sector decentralization. Their behavior, including decision autonomy, has largely been overlooked in the decentralization literature and may in fact help explain the varied effectiveness of these reforms. Building from this study, additional research is needed to connect variation in decision autonomy at the frontline to indicators of health system performance. Moreover, it is necessary to situate frontline decision autonomy within the multilevel systems that make up decentralized governance, and therefore more accurately characterize the re-distribution of autonomy across levels under decentralization reforms. These types of credible and specific findings will help strengthen the decentralized governance of health systems by suggesting potential changes in policy design and targeted efforts to support frontline health staff as they work to realize a sufficient degree of decision autonomy to respond effectively to the needs of their patients and communities.

## Supporting information

S1 File Online supporting information: autonomy survey questions, supplemental sensitivity and robustness analyses, and inclusivity in global research statement. (DOCX)
