# Supplementary material for: Frontline decision autonomy under decentralization: Evidence from health sector reform
Source: PLoS One. 2026 May 15;21(5):e0343736. doi: 10.1371/journal.pone.0343736 (PMC13178868; doi:10.1371/journal.pone.0343736)
Supplement: S1 File — (DOCX) [file pone.0343736.s001.docx]

Online Supporting Information for,

“Frontline Decision Autonomy under Decentralization:

Evidence from Health Sector Reform”

February 2024

**Appendix A – Autonomy Questions**

| Autonomía/Poder de Decisión y La Descentralización | |
| --- | --- |
| 1. ¿Cómo mira el nivel de descentralización en la administración de esta US? *How would you characterize the level of decentralization in the administration of the health center?* | 1. Muy centralizado (mucho control central) *Very centralized (a lot of central control)* 2. Centralizado (algún control central) *Centralized (some central control)* 3. Igual nivel de control local y control central *Equal level of municipal/local and central control* 4. Descentralizado (algún control local) *Decentralized (some municipal/local control)* 5. Muy descentralizado (mucho control local) *Very decentralized (a lot of municipal/local control)* 6. No sabe (NS) *Doesn’t know* 7. No quiere contestar (NC) *Doesn’t want to answer* |
| 1. ¿Cuál de las siguientes tres opciones refleja mejor el grado de autonomía/poder de decisión que tiene el personal de la US en cuanto a la planificación operativa y estratégica de la US? *Which of the following 3 options best reflects the degree of autonomy/decision power that the health center staff have in terms of the operational and strategic planning of the health center?* | 1. Ninguna autonomía/poder de decisión: el plan operativo anual (POA) no se desarrolla en la US, si no viene ya establecida por alguien de arriba (Región o gestor), y solo se la ejecuta *No autonomy/decision power: the annual operational plan is not developed in the health center, but rather comes alread established by someone from above (the Region or the decentralized managing organization), y it is only executed* 2. Algo de autonomía/poder de decisión: el plan operativo anual (POA) se desarrolla principalmente por la Región o el gestor, pero con algunas consultas, platicas, o reuniones con el personal de la US *Some autonomy/decision power: the annual operational plan is developed mainly by the Region or the decentralized managing organization, but with some consultation, discusions, or meetings with health center staff* 3. Mucha autonomía/poder de decisión: el plan operativo anual (POA) se desarrolla principalmente por el personal de la US, y basado en o reflejando en su mayoría las condiciones locales de la población en la área de cobertura de la US *Much autonomy/decision power: the annual operational plan is developed mainly by the health center staff, and based in or reflecting predominantly the local conditions of the population in the coverage área of the health center* 4. No sabe (NS) *Doesn’t know* 5. No quiere contestar (NC) *Doesn’t want to answer* |
| 1. ¿Cuál de las siguientes tres opciones refleja mejor el grado de autonomía/poder de decisión que tiene el personal de la US en cuanto a las finanzas y el presupuesto de la US? *Which of the following 3 options best reflects the degree of autonomy/decision power that the health center staff have in terms of the finances and budget of the health center?* | 1. Ninguna autonomía/poder de decisión: la US no cuenta con ninguna presupuesto ni otra fuente de fondos que puede manejar el personal, o no tiene el derecho ni la disposición para solicitar apoyo financiero de la Región, del Alcalde, del gestor, o de la comunidad (fondo solidario) *No autonomy/decision power: the health center does not have any Budget or other source of funds that the health center staff can manage, and does not have the right or disposition to solicit financial support from the Region, the Mayor, the managing organization, or the community (solidarity fund)* 2. Algo de autonomía/poder de decisión: la US cuenta con alguno apoyo financiero (podría ser por la Región, el Alcalde, el gestor, o la comunidad) donde pueden solicitar compras o rembolsar gastos no pre-aprobados de la US *Some autonomy/decision power: the health center has some financial supporty (could be from the Region, the Mayor, the decentralized managing organization, or teh community) where they can solicit purchases or reimburse non-pre-approved expenses of the health center* 3. Mucha autonomía/poder de decisión: la US cuenta con un fondo que el personal maneja internamente y que puede utilizar para compras o gastos de la US sin ninguna aprobación por la Región, el Alcalde, el gestor, o la comunidad *Much autonomy/decision power: the health center has a fund that staff manage internally and that they can utilize for purchases or expenses without approval by the Region, the Mayor, the decentralized managing organization, or the community* 4. No sabe (NS) *Doesn’t know* 5. No quiere contestar (NC) *Doesn’t want to answer* |
| 1. ¿Cuál de las siguientes tres opciones refleja mejor el grado de autonomía/poder de decisión que tiene el personal de la US en cuanto a los recursos humanos de la US? *Which of the following 3 options best reflects the degree of autonomy/decision power that the health center staff have in terms of the human resources of the health center?* | 1. Ninguna autonomía/poder de decisión: la US no tiene ningún control ni influencia sobre su personal o planilla, ni la disposición a solicitar cambios de personal (personal adicional, capacitación de personal débil, ni despido de personal ineficaz) *No autonomy/decision power: the health center does not have any control or influence about its staff, nor the disposition to solicit changes in staffing (additional staff, training for weak staff, or firing ineffective staff)* 2. Algo de autonomía/poder de decisión: la US tiene algún control e influencia sobre su personal o planilla a través de consultas o reuniones formales con la Región, el Alcalde, o el gestor, se sienten cómodos en expresar sus opiniones o solicitar cambios de personal, y a veces les toman en cuenta *Some autonomy/decision power: the health center has some control and influence over its staff through consultations or formal meetings where they feel comfortable in expressin their opinions or soliciting changes of staff, and are sometimes taken into account* 3. Mucha autonomía/poder de decisión: la US tiene casi todo el control sobre su personal o planilla que ellos manejan conjuntamente con la Región, el Alcalde, o el gestor, y la gran mayoría de decisiones con respecto al personal están tomadas por el mismo personal de la US *Much autonomy/decision power: the health center has almost complete control over its staff that they manage jointly with the Region, Mayor, decentralized managing organization, and the majority of decisions with respect to staff are taken by the same health center staff* 4. No sabe (NS) *Doesn’t know* 5. No quiere contestar (NC) *Doesn’t want to answer* |
| 1. ¿Cuál de las siguientes tres opciones refleja mejor el grado de autonomía/poder de decisión que tiene el personal de la US en cuanto a la organización y programación de servicios de la US? *Which of the following 3 options best reflects the degree of autonomy/decision power that the health center staff have in terms of the organization and programming of the services of the health center?* | 1. Ninguna autonomía/poder de decisión: la US no tiene ningún control ni influencia sobre sus servicios, ni cuales son ni como se proveen, y tampoco tienen la disposición a solicitar cambios en los servicios o sus organización para reflejar la condiciones de la población de su área de cobertura *No autonomy/decision power: the health centers has no control or influence over its services, neither which one nor how they are provided, y they also don’t have the disposition to solicit changes in the services or their organization to reflect the conditions of the population of its coverage area* 2. Algo de autonomía/poder de decisión: la US tiene algún control e influencia sobre sus servicios a través de consultas o reuniones formales con la Región, el Alcalde, o el gestor, se sienten cómodos en expresar sus opiniones o solicitar cambios de los servicios o su organización, y a veces les toman en cuenta *Some autonomy/decision power: the health center has some control and influence over its services through consultation or formal meetings with the Region, the Mayor, or the decentralized managing organization, they feel comfortable in expressing their opinions or to solicit changes to the services or their organization, and sometimes are taken into account* 3. Mucha autonomía/poder de decisión: la US tiene casi todo el control sobre sus servicios que ellos manejan conjuntamente con la Región, el Alcalde, o el gestor, y la gran mayoría de decisiones a los servicios y sus organización están tomadas por el mismo personal de la US *Much autonomy/decision power: the health center has almost total control over its services that they manage jointly with the Region, the Mayor, or the decentralized managing organization, and the great majority of the services or their organization are taken by the same staff of the health center* 4. No sabe (NS) *Doesn’t know* 5. No quiere contestar (NC) *Doesn’t want to answer* |

**Appendix B – Supplemental Analyses**

Table S2a: The Relationship between Decentralization Reform and Decision Autonomy Reported by Frontline Health Workers (Demographic Controls)

| *Decision Autonomy* | (1)  Decen. Percep. | (2)  Autonomy  Plan. | (3)  Autonomy Organiz. | (4)  Autonomy  HR. | (5)  Autonomy  Fin. |
| --- | --- | --- | --- | --- | --- |
| Decentralized (ref. cent.) | 0.85^**^ | -0.40^**^ | -0.27^**^ | -0.34^^^ | -0.21^^^ |
|  | (0.29) | (0.14) | (0.10) | (0.20) | (0.12) |
| Female | -0.08 | -0.08 | -0.07 | -0.10 | -0.13 |
|  | (0.14) | (0.09) | (0.08) | (0.09) | (0.10) |
| Education | 0.07 | 0.13^*^ | 0.08^*^ | 0.18^**^ | -0.01 |
|  | (0.09) | (0.06) | (0.04) | (0.06) | (0.05) |
| Age | 0.00 | -0.01 | -0.00 | -0.00 | 0.00 |
|  | (0.01) | (0.01) | (0.01) | (0.01) | (0.01) |
| Years in Health Sector | -0.00 | 0.01 | 0.01 | 0.02^ | 0.01 |
|  | (0.02) | (0.01) | (0.01) | (0.01) | (0.01) |
| Constant | 2.81^**^ | 2.13^**^ | 2.34^**^ | 1.30^**^ | 1.66^**^ |
|  | (0.47) | (0.30) | (0.29) | (0.41) | (0.28) |
| *N* | 526 | 581 | 584 | 582 | 574 |
| *R*^2^ | 0.14 | 0.08 | 0.06 | 0.10 | 0.05 |

Notes: Weighted OLS regression with clustered standard errors by municipality in parentheses; ^p<0.10, *p<0.05, **p<0.01

Table S2b: The Relationship between Decentralization Reform and Decision Autonomy Reported by Frontline Health Workers (Role Type Controls)

| *Decision Autonomy* | (1)  Decen. Percep. | (2)  Autonomy  Plan. | (3)  Autonomy Organiz. | (4)  Autonomy  HR. | (5)  Autonomy  Fin. |
| --- | --- | --- | --- | --- | --- |
| Decentralized (ref. cent.) | 0.97^**^ | -0.47^**^ | -0.34^**^ | -0.43^^^ | -0.14 |
|  | (0.25) | (0.15) | (0.10) | (0.23) | (0.11) |
| Nurse (ref. doctor) | -0.06 | -0.07 | -0.11 | -0.19 | 0.09 |
|  | (0.15) | (0.15) | (0.09) | (0.16) | (0.08) |
| Social Worker (ref. doctor) | -0.02 | 0.01 | -0.16 | -0.10 | -0.04 |
|  | (0.14) | (0.14) | (0.12) | (0.14) | (0.12) |
| Other Staff Type (ref. doctor) | 0.09 | -0.22 | -0.36^*^ | 0.01 | 0.13 |
|  | (0.20) | (0.21) | (0.14) | (0.21) | (0.13) |
| Constant | 2.97^**^ | 2.51^**^ | 2.82^**^ | 2.15^**^ | 1.51^**^ |
|  | (0.25) | (0.17) | (0.10) | (0.23) | (0.11) |
| *N* | 554 | 618 | 620 | 618 | 610 |
| *R*^2^ | 0.16 | 0.07 | 0.06 | 0.07 | 0.02 |

Notes: Weighted OLS regression with clustered standard errors by municipality in parentheses; ^p<0.10, *p<0.05, **p<0.01

Table S2b.i: The Relationship between Decentralization Reform and Decision Autonomy Reported by Frontline Health Workers (Role Type Controls Additional Category)

| *Decision Autonomy* | (1)  Decen. Percep. | (2)  Autonomy  Plan. | (5)  Autonomy Organiz. | (4)  Autonomy  HR. | (3)  Autonomy  Fin. |
| --- | --- | --- | --- | --- | --- |
| Decentralized (ref. cent.) | 0.93^**^ | -0.49^**^ | -0.35^**^ | -0.45^*^ | -0.16 |
|  | (0.24) | (0.14) | (0.10) | (0.22) | (0.11) |
| Nurse (ref. doctor) | -0.07 | -0.07 | -0.11 | -0.19 | 0.09 |
|  | (0.16) | (0.15) | (0.09) | (0.16) | (0.08) |
| Social Worker (ref. doctor) | -0.01 | 0.02 | -0.16 | -0.10 | -0.04 |
|  | (0.14) | (0.14) | (0.12) | (0.14) | (0.12) |
| Health Volunteer (ref. doctor) | -0.41 | -0.43^*^ | -0.43^**^ | -0.18 | -0.18 |
|  | (0.01) | (0.01) | (0.01) | (0.01) | (0.01) |
| Other Role (ref. doctor) | 1.00^*^ | 0.27^^^ | -0.17 | 0.45^*^ | 0.76^**^ |
|  | (0.45) | (0.16) | (0.18) | (0.20) | (0.29) |
| Constant | 2.99^**^ | 2.53^**^ | 2.83^**^ | 2.17^**^ | 1.52^**^ |
|  | (0.24) | (0.16) | (0.10) | (0.23) | (0.11) |
| *N* | 554 | 618 | 620 | 618 | 610 |
| *R*^2^ | 0.21 | 0.09 | 0.06 | 0.09 | 0.08 |

Notes: Weighted OLS regression, binary indicator of decentralization, role type controls models with clustered standard errors by municipality in parentheses; ^p<0.10, *p<0.05, **p<0.01

Table S3a: The Relationship between Decentralization by Organization Type and Decision Autonomy Reported by Frontline Health Workers (Demographic Controls)

| *Decision Autonomy* | (1)  Decen. Percep. | (2)  Autonomy  Plan. | (3)  Autonomy Organiz. | (4)  Autonomy  HR. | (5)  Autonomy  Fin. |
| --- | --- | --- | --- | --- | --- |
| Decent-MUNI (ref. cent.) | 0.74^*^ | -0.40^*^ | -0.58^**^ | -0.63^**^ | -0.37^**^ |
|  | (0.34) | (0.17) | (0.18) | (0.20) | (0.13) |
| Decent-ASSN (ref. cent.) | 1.00^**^ | -0.40^**^ | -0.12 | -0.23 | -0.34^**^ |
|  | (0.30) | (0.16) | (0.10) | (0.22) | (0.13) |
| Decent-NGO (ref. cent.) | 0.68^*^ | -0.41^^^ | -0.31^*^ | -0.31 | 0.14 |
|  | (0.31) | (0.21) | (0.14) | (0.23) | (0.14) |
| Female | -0.07 | -0.08 | -0.06 | -0.09 | -0.14 |
|  | (0.14) | (0.09) | (0.08) | (0.09) | (0.10) |
| Education | 0.06 | 0.13^*^ | 0.08^*^ | 0.18^**^ | 0.01 |
|  | (0.09) | (0.06) | (0.04) | (0.06) | (0.04) |
| Age | 0.00 | -0.01 | -0.00 | -0.00 | -0.00 |
|  | (0.01) | (0.01) | (0.01) | (0.01) | (0.01) |
| Years in Health Sector | -0.00 | 0.01 | 0.01^ | 0.02^ | 0.01 |
|  | (0.02) | (0.01) | (0.01) | (0.01) | (0.01) |
| Constant | 2.79^**^ | 2.13^**^ | 2.35^**^ | 1.31^**^ | 1.68^**^ |
|  | (0.46) | (0.30) | (0.29) | (0.40) | (0.28) |
| *N* | 526 | 581 | 584 | 582 | 574 |
| *R*^2^ | 0.15 | 0.08 | 0.11 | 0.13 | 0.11 |

Notes: Weighted OLS regression with clustered standard errors by municipality in parentheses; ^p<0.10, *p<0.05, **p<0.01

Table S3b: The Relationship between Decentralization Reform and Decision Autonomy Reported by Frontline Health Workers (Role Type Controls)

| *Decision Autonomy* | (1)  Decen. Percep. | (2)  Autonomy  Plan. | (3)  Autonomy Organiz. | (4)  Autonomy  HR. | (5)  Autonomy  Fin. |
| --- | --- | --- | --- | --- | --- |
| Decent-MUNI (ref. cent.) | 0.87^**^ | -0.47^**^ | -0.65^**^ | -0.68^**^ | -0.30^*^ |
|  | (0.30) | (0.18) | (0.18) | (0.24) | (0.12) |
| Decent-ASSN (ref. cent.) | 1.12^**^ | -0.46^**^ | -0.21^^^ | -0.32 | -0.26^*^ |
|  | (0.26) | (0.16) | (0.11) | (0.25) | (0.12) |
| Decent-NGO (ref. cent.) | 0.75^**^ | -0.50^*^ | -0.39^**^ | -0.47^^^ | 0.19 |
|  | (0.28) | (0.22) | (0.15) | (0.27) | (0.13) |
| Nurse (ref. doctor) | -0.04 | -0.06 | -0.08 | -0.16 | 0.09 |
|  | (0.16) | (0.15) | (0.08) | (0.16) | (0.08) |
| Social Worker (ref. doctor) | 0.03 | 0.02 | -0.10 | -0.05 | -0.05 |
|  | (0.14) | (0.14) | (0.11) | (0.14) | (0.12) |
| Other Staff Type (ref. doctor) | 0.15 | -0.21 | -0.32* | 0.04 | 0.06 |
|  | (0.19) | (0.21) | (0.14) | (0.21) | (0.14) |
| Constant | 2.93^**^ | 2.51^**^ | 2.79^**^ | 2.13^**^ | 1.52^**^ |
|  | (0.25) | (0.16) | (0.10) | (0.23) | (0.11) |
| *N* | 554 | 618 | 620 | 618 | 610 |
| *R*^2^ | 0.18 | 0.07 | 0.10 | 0.09 | 0.08 |

Notes: Weighted OLS regression with clustered standard errors by municipality in parentheses; ^p<0.10, *p<0.05, **p<0.01

Table S3b.i: The Relationship between Decentralization Reform and Decision Autonomy Reported by Frontline Health Workers (Role Type Controls Additional Category)

| *Decision Autonomy* | (1)  Decen. Percep. | (2)  Autonomy  Plan. | (5)  Autonomy Organiz. | (4)  Autonomy  HR. | (3)  Autonomy  Fin. |
| --- | --- | --- | --- | --- | --- |
| Decent-MUNI (ref. cent.) | 0.84^**^ | -0.49^**^ | -0.366^**^ | -0.70^**^ | -0.32^**^ |
|  | (0.30) | (0.17) | (0.18) | (0.24) | (0.12) |
| Decent-ASSN (ref. cent.) | 1.08^**^ | -0.49^**^ | -0.21^^^ | -0.34 | -0.29^*^ |
|  | (0.26) | (0.15) | (0.11) | (0.24) | (0.11) |
| Decent-NGO (ref. cent.) | 0.74^**^ | -0.51^*^ | -0.39^**^ | -0.48^^^ | 0.18 |
|  | (0.26) | (0.21) | (0.15) | (0.26) | (0.13) |
| Nurse (ref. doctor) | -0.05 | -0.07 | -0.08 | -0.16 | 0.08 |
|  | (0.16) | (0.15) | (0.08) | (0.16) | (0.08) |
| Social Worker (ref. doctor) | 0.03 | 0.02 | -0.10 | -0.05 | -0.05 |
|  | (0.14) | (0.14) | (0.11) | (0.14) | (0.12) |
| Health Volunteer (ref. doctor) | -0.35 | -0.43^*^ | -0.40^**^ | -0.15 | -0.26^^^ |
|  | (0.32) | (0.21) | (0.14) | (0.20) | (0.14) |
| Other Role (ref. doctor) | 1.04^*^ | 0.28^^^ | -0.15 | 0.46^*^ | 0.72^*^ |
|  | (0.44) | (0.16) | (0.19) | (0.20) | (0.31) |
| Constant | 2.96^**^ | 2.53^**^ | 2.79^**^ | 2.14^**^ | 1.54^**^ |
|  | (0.24) | (0.16) | (0.10) | (0.23) | (0.11) |
| *N* | 554 | 618 | 620 | 618 | 610 |
| *R*^2^ | 0.22 | 0.09 | 0.10 | 0.11 | 0.14 |

Notes: Weighted OLS regression, binary indicator of decentralization, role type controls models with clustered standard errors by municipality in parentheses; ^p<0.10, *p<0.05, **p<0.01

Table S4b: The Relationship between Decentralization Reform and Decision Autonomy among Role Types Reported by Frontline Health Workers

| *Decision Autonomy* | (1)  Decen. Percep. | (2)  Autonomy  Plan. | (3)  Autonomy Organiz. | (4)  Autonomy  HR. | (5)  Autonomy  Fin. |
| --- | --- | --- | --- | --- | --- |
| Decentralized (ref. cent.) | 1.36^**^ | -0.86^**^ | -0.18 | -0.57 | -0.20 |
|  | (0.32) | (0.19) | (0.20) | (0.36) | (0.23) |
| Nurse (ref. doctor) | 0.29 | -0.37^*^ | 0.05 | -0.30 | 0.06 |
|  | (0.41) | (0.17) | (0.20) | (0.39) | (0.21) |
| Social Worker (ref. doctor) | -0.27 | -0.75 | 0.07 | -0.41 | -0.47^*^ |
|  | (0.53) | (0.48) | (0.22) | (0.58) | (0.23) |
| Other Staff Type (ref. doctor) | 0.53 | -0.57^^^ | -0.26 | -0.11 | 0.05 |
|  | (0.38) | (0.32) | (0.28) | (0.48) | (0.29) |
| Decent. X Nurse | -0.42 | 0.37 | -0.20 | 0.13 | 0.02 |
|  | (0.44) | (0.25) | (0.22) | (0.43) | (0.22) |
| Decent. X Social Worker | 0.23 | 0.86^^^ | -0.27 | 0.34 | 0.46^^^ |
|  | (0.55) | (0.50) | (0.25) | (0.60) | (0.26) |
| Decent. X Other Staff Type | -0.69 | 0.49 | -0.02 | 0.16 | 0.13 |
|  | (0.45) | (0.38) | (0.32) | (0.52) | (0.32) |
| Constant | 2.63^**^ | 2.83^**^ | 2.69^**^ | 2.27^**^ | 1.56^**^ |
|  | (0.29) | (0.11) | (0.16) | (0.32) | (0.21) |
| *N* | 554 | 618 | 620 | 618 | 610 |
| *R*^2^ | 0.17 | 0.08 | 0.06 | 0.07 | 0.03 |

Notes: Weighted OLS regression, binary indicator of decentralization, role type controls models with clustered standard errors by municipality in parentheses; ^p<0.10, *p<0.05, **p<0.01

Table S5a: The Relationship between Decentralization Reform, Capacity, Resources, and Accountability and Decision Autonomy Reported by Frontline Health Workers (Demographic Controls)

| *Decision Autonomy* | (1)  Auto  Plan | (2)  Auto  Prog | (3)  Auto  HR | (4)  Auto  Fin |
| --- | --- | --- | --- | --- |
| *Administrative Form* |  |  |  |  |
| Dec-MUNI (ref. cent.) | -0.47^**^ | -0.73^**^ | -0.67^**^ | -0.36^*^ |
|  | (0.16) | (0.17) | (0.17) | (0.14) |
| Dec-ASSN (ref. cent.) | -0.41^**^ | -0.23^^^ | -0.31^^^ | -0.33^**^ |
|  | (0.14) | (0.13) | (0.18) | (0.13) |
| Dec-NGO (ref. cent.) | -0.47^*^ | -0.45^**^ | -0.44^*^ | 0.10 |
|  | (0.20) | (0.15) | (0.20) | (0.14) |
| *Capacity* |  |  |  |  |
| Yrs in Hlth. Sector | 0.01 | 0.01 | 0.01 | 0.01 |
|  | (0.01) | (0.01) | (0.01) | (0.01) |
| Perc. Staff Leadership | 0.19^*^ | 0.18^**^ | 0.04 | 0.07 |
|  | (0.08) | (0.06) | (0.07) | (0.07) |
| *Resources* |  |  |  |  |
| Perc. HC Resources | 0.17^**^ | 0.10^*^ | 0.29^**^ | 0.01 |
|  | (0.05) | (0.05) | (0.05) | (0.04) |
| Perc. Freq. Hlth. Use | -0.05 | -0.03 | -0.07 | 0.13 |
|  | (0.07) | (0.06) | (0.06) | (0.10) |
| *Accountability* |  |  |  |  |
| Tot. Evaluation Visits | -0.00 | 0.01 | -0.01 | -0.01 |
|  | (0.01) | (0.01) | (0.01) | (0.01) |
| Tot. Support Visits | 0.01 | 0.00 | 0.02^^^ | 0.01^*^ |
|  | (0.01) | (0.01) | (0.01) | (0.01) |
| *Demographic Controls* |  |  |  |  |
| Age | -0.00 | -0.00 | -0.00 | -0.00 |
|  | (0.01) | (0.01) | (0.01) | (0.01) |
| Female | -0.04 | -0.01 | -0.05 | -0.14 |
|  | (0.08) | (0.08) | (0.08) | (0.10) |
| Education | 0.08 | 0.05 | 0.11^^^ | -0.02 |
|  | (0.06) | (0.04) | (0.06) | (0.05) |
| Constant | 1.06^**^ | 1.44^**^ | 0.77^^^ | 1.03^*^ |
|  | (0.40) | (0.33) | (0.46) | (0.43) |
| *N* | 574 | 577 | 575 | 567 |
| *R*^2^ | 0.16 | 0.17 | 0.24 | 0.15 |

Notes: Weighted OLS regression with clustered standard errors by municipality in parentheses; **p<0.01, *p<0.05, ^p<0.10

Table S5b: The Relationship between Decentralization Reform, Capacity, Resources, and Accountability and Decision Autonomy Reported by Frontline Health Workers (Role Type Controls)

| *Decision Autonomy* | (1)  Auto  Plan | (2)  Auto  Prog | (3)  Auto  HR | (4)  Auto  Fin |
| --- | --- | --- | --- | --- |
| *Administrative Form* |  |  |  |  |
| Dec-MUNI (ref. cent.) | -0.48^**^ | -0.76^**^ | -0.63^**^ | -0.31^*^ |
|  | (0.15) | (0.17) | (0.18) | (0.13) |
| Dec-ASSN (ref. cent.) | -0.42^**^ | -0.28^*^ | -0.30^^^ | -0.28^*^ |
|  | (0.14) | (0.13) | (0.18) | (0.12) |
| Dec-NGO (ref. cent.) | -0.49^*^ | -0.48^**^ | -0.48^*^ | 0.14 |
|  | (0.20) | (0.15) | (0.20) | (0.13) |
| *Capacity* |  |  |  |  |
| Yrs in Hlth. Sector | 0.01 | 0.01 | 0.01^*^ | 0.00 |
|  | (0.01) | (0.00) | (0.01) | (0.01) |
| Perc. Staff Leadership | 0.21^*^ | 0.18^**^ | 0.08 | 0.05 |
|  | (0.08) | (0.06) | (0.08) | (0.06) |
| *Resources* |  |  |  |  |
| Perc. HC Resources | 0.19^**^ | 0.10^*^ | 0.30^**^ | 0.02 |
|  | (0.04) | (0.04) | (0.05) | (0.04) |
| Perc. Freq. Hlth. Use | -0.04 | -0.02 | -0.12^*^ | 0.15 |
|  | (0.07) | (0.05) | (0.06) | (0.11) |
| *Accountability* |  |  |  |  |
| Tot. Evaluation Visits | -0.01 | 0.01 | -0.01 | -0.00 |
|  | (0.01) | (0.01) | (0.01) | (0.01) |
| Tot. Support Visits | 0.01 | 0.00 | 0.02^*^ | 0.01^**^ |
|  | (0.01) | (0.01) | (0.01) | (0.01) |
| *Role Type Controls* |  |  |  |  |
| Nurse (ref. doc.) | -0.11 | -0.07 | -0.21^^^ | 0.13 |
|  | (0.12) | (0.09) | (0.12) | (0.09) |
| Soc. Worker (ref. doc.) | -0.07 | -0.12 | -0.11 | -0.03 |
|  | (0.13) | (0.11) | (0.12) | (0.13) |
| Other Staff (ref. doc.) | -0.23 | -0.28^*^ | 0.03 | 0.06 |
|  | (0.15) | (0.11) | (0.12) | (0.12) |
| Constant | 1.19^**^ | 1.67^**^ | 1.14^**^ | 0.65 |
|  | (0.32) | (0.30) | (0.37) | (0.50) |
| *N* | 611 | 613 | 611 | 603 |
| *R*^2^ | 0.17 | 0.19 | 0.25 | 0.13 |

Notes: Weighted OLS regression with clustered standard errors by municipality in parentheses; **p<0.01, *p<0.05, ^p<0.10

Table S5c.i: The Relationship between Decentralization by Organization Type and Decision Reported by Frontline Health Workers, **Decen Percep**, All Models

| *Decision Autonomy* | (1)  Decen Percep | (2)  Decen Percep | (3)  Decen Percep | (4)  Decen Percep | (5)  Decen Percep | (6)  Decen Percep | (7)  Decen Percep | (8)  Decen Percep | (9)  Decen Percep |
| --- | --- | --- | --- | --- | --- | --- | --- | --- | --- |
| *Administrative Form* |  |  |  |  |  |  |  |  |  |
| Dec-MUNI (ref. cent.) | 0.84^**^ |  |  |  |  |  | 0.74^*^ | 0.80^**^ | 0.78^**^ |
|  | (0.30) |  |  |  |  |  | (0.30) | (0.26) | (0.29) |
| Dec-ASSN (ref. cent.) | 1.10^***^ |  |  |  |  |  | 1.05^***^ | 1.08^***^ | 1.09^***^ |
|  | (0.26) |  |  |  |  |  | (0.25) | (0.22) | (0.25) |
| Dec-NGO (ref. cent.) | 0.75^**^ |  |  |  |  |  | 0.68^*^ | 0.69^**^ | 0.71^**^ |
|  | (0.29) |  |  |  |  |  | (0.28) | (0.25) | (0.27) |
| *Capacity* |  |  |  |  |  |  |  |  |  |
| Yrs in Hlth. Sector |  | -0.02 |  |  |  |  | -0.01 | -0.01 | -0.01 |
|  |  | (0.01) |  |  |  |  | (0.01) | (0.01) | (0.01) |
| Perc. Staff Leadership |  | -0.07 |  |  |  |  | 0.05 | (0.02) | 0.06 |
|  |  | (0.10) |  |  |  |  | (0.11) | (0.09) | (0.11) |
| *Accountability* |  |  |  |  |  |  |  |  |  |
| Tot. Evaluation Visits |  |  | 0.03^^^ |  |  |  | -0.00 | -0.00 | -0.00 |
|  |  |  | (0.01) |  |  |  | (0.01) | (0.01) | (0.01) |
| Tot. Support Visits |  |  | 0.01 |  |  |  | 0.00 | 0.01 | 0.00 |
|  |  |  | (0.01) |  |  |  | (0.01) | (0.01) | (0.01) |
| *Resources* |  |  |  |  |  |  |  |  |  |
| Perc. HC Resources |  |  |  | 0.21^**^ |  |  | 0.22^^^ | 0.21^^^ | 0.20^*^ |
|  |  |  |  | (0.06) |  |  | (0.11) | (0.12) | (0.10) |
| Perc. Freq. Hlth. Use |  |  |  | 0.08 |  |  | 0.14 | 0.15 | 0.12 |
|  |  |  |  | (0.12) |  |  | (0.11) | (0.12) | (0.10) |
| *Demographic Controls* |  |  |  |  |  |  |  |  |  |
| Age |  |  |  |  | -0.01 |  | 0.00 |  | 0.00 |
|  |  |  |  |  | (0.01) |  | (0.01) |  | (0.01) |
| Female |  |  |  |  | -0.16 |  | -0.04 |  | -0.04 |
|  |  |  |  |  | (0.12) |  | (0.11) |  | (0.14) |
| Education |  |  |  |  | 0.11 |  | -0.00 |  | 0.06 |
|  |  |  |  |  | (0.11) |  | (0.07) |  | (0.09) |
| *Role Type Controls* |  |  |  |  |  |  |  |  |  |
| Nurse (ref. doc.) |  |  |  |  |  | -0.25 |  | -0.02 | 0.09 |
|  |  |  |  |  |  | (0.18) |  | (0.12) | (0.19) |
| Soc. Worker (ref. doc.) |  |  |  |  |  | 0.08 |  | -0.01 | 0.04 |
|  |  |  |  |  |  | (0.17) |  | (0.13) | (0.17) |
| Other Staff (ref. doc.) |  |  |  |  |  | -0.40^^^ |  | 0.11 | 0.38 |
|  |  |  |  |  |  | (0.22) |  | (0.18) | (0.30) |
| Constant | 2.95^***^ | 4.06^***^ | 3.26^***^ | 2.75^***^ | 3.81^***^ | 3.79^***^ | 1.69^*^ | 1.84^*^ | 1.47^^^ |
|  | (0.24) | (0.45) | (0.17) | (0.46) | (0.65) | (0.15) | (0.69) | (0.72) | (0.85) |
| *N* | 554 | 552 | 554 | 550 | 526 | 554 | 520 | 548 | 520 |
| *R*^2^ | 0.17 | 0.02 | 0.06 | 0.03 | 0.03 | 0.02 | 0.20 | 0.22 | 0.20 |

Notes: Weighted OLS regression with clustered standard errors by municipality in parentheses; ***p<0.001, **p<0.01, *p<0.05, ^p<0.10

Table S5c.ii: The Relationship between Decentralization by Organization Type and Decision Reported by Frontline Health Workers, **Auto Plan**, All Models

| *Decision Autonomy* | (1)  Auto Plan | (2)  Auto Plan | (3)  Auto Plan | (4)  Auto Plan | (5)  Auto Plan | (6)  Auto Plan | (7)  Auto Plan | (8)  Auto Plan | (9)  Auto Plan |
| --- | --- | --- | --- | --- | --- | --- | --- | --- | --- |
| *Administrative Form* |  |  |  |  |  |  |  |  |  |
| Dec-MUNI (ref. cent.) | -0.40^*^ |  |  |  |  |  | -0.47^**^ | -0.48^**^ | -0.47^**^ |
|  | (0.18) |  |  |  |  |  | (0.16) | (0.15) | (0.17) |
| Dec-ASSN (ref. cent.) | -0.40^*^ |  |  |  |  |  | -0.41^**^ | -0.42^**^ | -0.43^**^ |
|  | (0.17) |  |  |  |  |  | (0.14) | (0.14) | (0.15) |
| Dec-NGO (ref. cent.) | -0.45^*^ |  |  |  |  |  | -0.47^*^ | -0.49^*^ | -0.47^*^ |
|  | (0.23) |  |  |  |  |  | (0.20) | (0.20) | (0.20) |
| *Capacity* |  |  |  |  |  |  |  |  |  |
| Yrs in Hlth. Sector |  | 0.01^^^ |  |  |  |  | 0.01 | 0.01 | 0.01 |
|  |  | (0.01) |  |  |  |  | (0.01) | (0.01) | (0.01) |
| Perc. Staff Leadership |  | 0.28^**^ |  |  |  |  | 0.19^*^ | 0.21^*^ | 0.19^*^ |
|  |  | (0.10) |  |  |  |  | (0.08) | (0.08) | (0.09) |
| *Accountability* |  |  |  |  |  |  |  |  |  |
| Tot. Evaluation Visits |  |  | -0.02^*^ |  |  |  | -0.00 | -0.01 | -0.00 |
|  |  |  | (0.01) |  |  |  | (0.01) | (0.01) | (0.01) |
| Tot. Support Visits |  |  | 0.02^^^ |  |  |  | 0.01 | 0.01 | 0.01 |
|  |  |  | (0.01) |  |  |  | (0.01) | (0.01) | (0.01) |
| *Resources* |  |  |  |  |  |  |  |  |  |
| Perc. HC Resources |  |  |  | 0.23^***^ |  |  | 0.17^**^ | 0.19^***^ | 0.17^***^ |
|  |  |  |  | (0.07) |  |  | (0.05) | (0.04) | (0.05) |
| Perc. Freq. Hlth. Use |  |  |  | 0.00 |  |  | -0.05 | -0.04 | -0.04 |
|  |  |  |  | (0.06) |  |  | (0.07) | (0.07) | (0.07) |
| *Demographic Controls* |  |  |  |  |  |  |  |  |  |
| Age |  |  |  |  | 0.01 |  | -0.00 |  | -0.01 |
|  |  |  |  |  | (0.01) |  | (0.01) |  | (0.01) |
| Female |  |  |  |  | -0.02 |  | -0.04 |  | -0.07 |
|  |  |  |  |  | (0.09) |  | (0.08) |  | (0.09) |
| Education |  |  |  |  | 0.13^*^ |  | 0.08 |  | 0.03 |
|  |  |  |  |  | (0.06) |  | (0.06) |  | (0.07) |
| *Role Type Controls* |  |  |  |  |  |  |  |  |  |
| Nurse (ref. doc.) |  |  |  |  |  | 0.05 |  | -0.11 | -0.12 |
|  |  |  |  |  |  | (0.17) |  | (0.12) | (0.15) |
| Soc. Worker (ref. doc.) |  |  |  |  |  | -0.04 |  | -0.07 | -0.18 |
|  |  |  |  |  |  | (0.15) |  | (0.13) | (0.16) |
| Other Staff (ref. doc.) |  |  |  |  |  | 0.04 |  | -0.23 | -0.24 |
|  |  |  |  |  |  | (0.27) |  | (0.15) | (0.17) |
| Constant | 2.41^***^ | 0.84^*^ | 2.23^***^ | 1.48^***^ | 1.37^***^ | 2.12^***^ | 1.06^**^ | 1.19^***^ | 1.40^**^ |
|  | (0.13) | (0.41) | (0.13) | (0.31) | (0.34) | (0.15) | (0.40) | (0.32) | (0.49) |
| *N* | 618 | 615 | 618 | 614 | 581 | 618 | 574 | 611 | 574 |
| *R*^2^ | 0.06 | 0.08 | 0.03 | 0.06 | 0.02 | 0.00 | 0.16 | 0.17 | 0.17 |

Notes: Weighted OLS regression with clustered standard errors by municipality in parentheses; ***p<0.001, **p<0.01, *p<0.05, ^p<0.10

Table S5c.iii: The Relationship between Decentralization by Organization Type and Decision Reported by Frontline Health Workers, **Auto Fin**, All Models

| *Decision Autonomy* | (1)  Auto Fin | (2)  Auto Fin | (3)  Auto Fin | (4)  Auto Fin | (5)  Auto Fin | (6)  Auto Fin | (7)  Auto Fin | (8)  Auto Fin | (9)  Auto Fin |
| --- | --- | --- | --- | --- | --- | --- | --- | --- | --- |
| *Administrative Form* |  |  |  |  |  |  |  |  |  |
| Dec-MUNI (ref. cent.) | -0.34^**^ |  |  |  |  |  | -0.36^*^ | -0.31^*^ | -0.30^*^ |
|  | (0.12) |  |  |  |  |  | (0.14) | (0.13) | (0.14) |
| Dec-ASSN (ref. cent.) | -0.30^*^ |  |  |  |  |  | -0.33^**^ | -0.28^*^ | -0.29^*^ |
|  | (0.12) |  |  |  |  |  | (0.13) | (0.12) | (0.12) |
| Dec-NGO (ref. cent.) | 0.15 |  |  |  |  |  | 0.10 | 0.14 | 0.16 |
|  | (0.14) |  |  |  |  |  | (0.14) | (0.13) | (0.13) |
| *Capacity* |  |  |  |  |  |  |  |  |  |
| Yrs in Hlth. Sector |  | 0.01 |  |  |  |  | 0.01 | 0.00 | 0.01 |
|  |  | (0.01) |  |  |  |  | (0.01) | (0.01) | (0.01) |
| Perc. Staff Leadership |  | 0.08 |  |  |  |  | 0.07 | 0.05 | 0.07 |
|  |  | (0.07) |  |  |  |  | (0.07) | (0.06) | (0.07) |
| *Accountability* |  |  |  |  |  |  |  |  |  |
| Tot. Evaluation Visits |  |  | -0.01^^^ |  |  |  | -0.01 | -0.00 | -0.01 |
|  |  |  | (0.01) |  |  |  | (0.01) | (0.01) | (0.01) |
| Tot. Support Visits |  |  | 0.02^*^ |  |  |  | 0.01^*^ | 0.01^**^ | 0.01^*^ |
|  |  |  | (0.01) |  |  |  | (0.01) | (0.01) | (0.01) |
| *Resources* |  |  |  |  |  |  |  |  |  |
| Perc. HC Resources |  |  |  | 0.04 |  |  | 0.01 | 0.02 | -0.00 |
|  |  |  |  | (0.05) |  |  | (0.04) | (0.04) | (0.04) |
| Perc. Freq. Hlth. Use |  |  |  | 0.17 |  |  | 0.13 | 0.15 | 0.13 |
|  |  |  |  | (0.12) |  |  | (0.10) | (0.11) | (0.09) |
| *Demographic Controls* |  |  |  |  |  |  |  |  |  |
| Age |  |  |  |  | 0.01 |  | -0.00 |  | -0.00 |
|  |  |  |  |  | (0.01) |  | (0.01) |  | (0.04) |
| Female |  |  |  |  | -0.10 |  | -0.14 |  | -0.27^**^ |
|  |  |  |  |  | (0.09) |  | (0.10) |  | (0.09) |
| Education |  |  |  |  | -0.00 |  | -0.02 |  | 0.03 |
|  |  |  |  |  | (0.05) |  | (0.05) |  | (0.04) |
| *Role Type Controls* |  |  |  |  |  |  |  |  |  |
| Nurse (ref. doc.) |  |  |  |  |  | 0.12 |  | 0.13 | 0.24^*^ |
|  |  |  |  |  |  | (0.08) |  | (0.09) | (0.10) |
| Soc. Worker (ref. doc.) |  |  |  |  |  | -0.06 |  | -0.03 | -0.11 |
|  |  |  |  |  |  | (0.12) |  | (0.13) | (0.14) |
| Other Staff (ref. doc.) |  |  |  |  |  | 0.20 |  | 0.06 | 0.25 |
|  |  |  |  |  |  | (0.16) |  | (0.12) | (0.16) |
| Constant | 1.60^***^ | 1.07^***^ | 1.49^***^ | 0.79^^^ | 1.24^***^ | 1.39^***^ | 1.03^*^ | 0.65 | 0.84^^^ |
|  | (0.10) | (0.29) | (0.08) | (0.44) | (0.32) | (0.08) | (0.43) | (0.50) | (0.46) |
| *N* | 610 | 607 | 610 | 606 | 574 | 610 | 567 | 603 | 567 |
| *R*^2^ | 0.08 | 0.02 | 0.04 | 0.04 | 0.02 | 0.02 | 0.15 | 0.13 | 0.17 |

Notes: Weighted OLS regression with clustered standard errors by municipality in parentheses; ***p<0.001, **p<0.01, *p<0.05, ^p<0.10

Table S5c.iv: The Relationship between Decentralization by Organization Type and Decision Reported by Frontline Health Workers, **Auto HR**, All Models

| *Decision Autonomy* | (1)  Auto HR | (2)  Auto HR | (3)  Auto HR | (4)  Auto HR | (5)  Auto HR | (6)  Auto HR | (7)  Auto HR | (8)  Auto HR | (9)  Auto HR |
| --- | --- | --- | --- | --- | --- | --- | --- | --- | --- |
| *Administrative Form* |  |  |  |  |  |  |  |  |  |
| Dec-MUNI (ref. cent.) | -0.69^**^ |  |  |  |  |  | -0.67^***^ | -0.63^***^ | -0.63^***^ |
|  | (0.26) |  |  |  |  |  | (0.17) | (0.18) | (0.18) |
| Dec-ASSN (ref. cent.) | -0.32 |  |  |  |  |  | -0.31^^^ | -0.30^^^ | -0.27 |
|  | (0.27) |  |  |  |  |  | (0.18) | (0.18) | (0.18) |
| Dec-NGO (ref. cent.) | -0.45 |  |  |  |  |  | -0.44^*^ | -0.48^*^ | -0.42^*^ |
|  | (0.29) |  |  |  |  |  | (0.20) | (0.20) | (0.20) |
| *Capacity* |  |  |  |  |  |  |  |  |  |
| Yrs in Hlth. Sector |  | 0.02^^^ |  |  |  |  | 0.01 | 0.01^*^ | 0.02^^^ |
|  |  | (0.01) |  |  |  |  | (0.01) | (0.01) | (0.01) |
| Perc. Staff Leadership |  | 0.14 |  |  |  |  | 0.04 | 0.08 | 0.05 |
|  |  | (0.12) |  |  |  |  | (0.07) | (0.08) | (0.06) |
| *Accountability* |  |  |  |  |  |  |  |  |  |
| Tot. Evaluation Visits |  |  | -0.03^*^ |  |  |  | -0.01 | -0.01 | -0.01 |
|  |  |  | (0.01) |  |  |  | (0.01) | (0.01) | (0.01) |
| Tot. Support Visits |  |  | 0.03^*^ |  |  |  | 0.02^^^ | 0.02^*^ | 0.02^^^ |
|  |  |  | (0.01) |  |  |  | (0.01) | (0.01) | (0.01) |
| *Resources* |  |  |  |  |  |  |  |  |  |
| Perc. HC Resources |  |  |  | 0.35^***^ |  |  | 0.29^***^ | 0.30^***^ | 0.28^***^ |
|  |  |  |  | (0.08) |  |  | (0.05) | (0.05) | (0.05) |
| Perc. Freq. Hlth. Use |  |  |  | -0.06 |  |  | -0.07 | -0.12^*^ | -0.10 |
|  |  |  |  | (0.06) |  |  | (0.06) | (0.06) | (0.06) |
| *Demographic Controls* |  |  |  |  |  |  |  |  |  |
| Age |  |  |  |  | 0.01^^^ |  | -0.00 |  | -0.00 |
|  |  |  |  |  | (0.01) |  | (0.01) |  | (0.01) |
| Female |  |  |  |  | -0.04 |  | -0.05 |  | -0.02 |
|  |  |  |  |  | (0.10) |  | (0.08) |  | (0.09) |
| Education |  |  |  |  | 0.19^**^ |  | 0.11^^^ |  | 0.16^^^ |
|  |  |  |  |  | (0.07) |  | (0.06) |  | (0.09) |
| *Role Type Controls* |  |  |  |  |  |  |  |  |  |
| Nurse (ref. doc.) |  |  |  |  |  | -0.09 |  | -0.21^^^ | 0.02 |
|  |  |  |  |  |  | (0.18) |  | (0.12) | (0.16) |
| Soc. Worker (ref. doc.) |  |  |  |  |  | -0.15 |  | -0.11 | 0.05 |
|  |  |  |  |  |  | (0.15) |  | (0.12) | (0.15) |
| Other Staff (ref. doc.) |  |  |  |  |  | 0.23 |  | 0.03 | 0.31 |
|  |  |  |  |  |  | (0.29) |  | (0.12) | (0.24) |
| Constant | 2.03^***^ | 0.95^^^ | 1.80^***^ | 0.97^**^ | 0.53 | 1.79^***^ | 0.77^^^ | 1.14^**^ | 0.56 |
|  | (0.24) | (0.51) | (0.21) | (0.32) | (0.39) | (0.15) | (0.46) | (0.37) | (0.50) |
| *N* | 618 | 615 | 618 | 614 | 582 | 618 | 575 | 611 | 575 |
| *R*^2^ | 0.08 | 0.07 | 0.04 | 0.11 | 0.05 | 0.02 | 0.24 | 0.25 | 0.25 |

Notes: Weighted OLS regression with clustered standard errors by municipality in parentheses; ***p<0.001, **p<0.01, *p<0.05, ^p<0.10

Table S5c.v: The Relationship between Decentralization by Organization Type and Decision Reported by Frontline Health Workers, **Auto Prog**, All Models

| *Decision Autonomy* | (1)  Auto Prog | (2)  Auto Prog | (3)  Auto Prog | (4)  Auto Prog | (5)  Auto Prog | (6)  Auto Prog | (7)  Auto Prog | (8)  Auto Prog | (9)  Auto Prog |
| --- | --- | --- | --- | --- | --- | --- | --- | --- | --- |
| *Administrative Form* |  |  |  |  |  |  |  |  |  |
| Dec-MUNI (ref. cent.) | -0.59^**^ |  |  |  |  |  | -0.73^***^ | -0.76^***^ | -0.75^***^ |
|  | (0.18) |  |  |  |  |  | (0.17) | (0.17) | (0.17) |
| Dec-ASSN (ref. cent.) | -0.15 |  |  |  |  |  | -0.23^^^ | -0.28^*^ | -0.26^*^ |
|  | (0.12) |  |  |  |  |  | (0.13) | (0.13) | (0.12) |
| Dec-NGO (ref. cent.) | -0.35^*^ |  |  |  |  |  | -0.45^**^ | -0.48^**^ | -0.46^**^ |
|  | (0.16) |  |  |  |  |  | (0.15) | (0.15) | (0.14) |
| *Capacity* |  |  |  |  |  |  |  |  |  |
| Yrs in Hlth. Sector |  | 0.01^**^ |  |  |  |  | 0.01 | 0.01 | 0.00 |
|  |  | (0.00) |  |  |  |  | (0.01) | (0.00) | (0.01) |
| Perc. Staff Leadership |  | 0.18^*^ |  |  |  |  | 0.18^**^ | 0.18^**^ | 0.16^*^ |
|  |  | (0.07) |  |  |  |  | (0.06) | (0.06) | (0.07) |
| *Accountability* |  |  |  |  |  |  |  |  |  |
| Tot. Evaluation Visits |  |  | -0.00 |  |  |  | 0.01 | 0.01 | 0.01 |
|  |  |  | (0.01) |  |  |  | (0.01) | (0.01) | (0.01) |
| Tot. Support Visits |  |  | 0.01 |  |  |  | 0.00 | 0.00 | 0.01 |
|  |  |  | (0.01) |  |  |  | (0.01) | (0.01) | (0.01) |
| *Resources* |  |  |  |  |  |  |  |  |  |
| Perc. HC Resources |  |  |  | 0.15^**^ |  |  | 0.10^*^ | 0.10^*^ | 0.11^*^ |
|  |  |  |  | (0.05) |  |  | (0.05) | (0.04) | (0.05) |
| Perc. Freq. Hlth. Use |  |  |  | -0.01 |  |  | -0.03 | -0.02 | -0.01 |
|  |  |  |  | (0.05) |  |  | (0.06) | (0.05) | (0.06) |
| *Demographic Controls* |  |  |  |  |  |  |  |  |  |
| Age |  |  |  |  | 0.01^^^ |  | -0.00 |  | 0.00 |
|  |  |  |  |  | (0.00) |  | (0.01) |  | (0.01) |
| Female |  |  |  |  | -0.03 |  | -0.01 |  | -0.06 |
|  |  |  |  |  | (0.09) |  | (0.08) |  | (0.09) |
| Education |  |  |  |  | 0.09^*^ |  | 0.05 |  | 0.02 |
|  |  |  |  |  | (0.04) |  | (0.04) |  | (0.05) |
| *Role Type Controls* |  |  |  |  |  |  |  |  |  |
| Nurse (ref. doc.) |  |  |  |  |  | -0.03 |  | -0.07 | 0.08 |
|  |  |  |  |  |  | (0.09) |  | (0.09) | (0.11) |
| Soc. Worker (ref. doc.) |  |  |  |  |  | -0.20^^^ |  | -0.12 | -0.03 |
|  |  |  |  |  |  | (0.12) |  | (0.11) | (0.14) |
| Other Staff (ref. doc.) |  |  |  |  |  | -0.18 |  | -0.28^*^ | -0.20 |
|  |  |  |  |  |  | (0.19) |  | (0.11) | (0.14) |
| Constant | 2.64^***^ | 1.58^***^ | 2.43^***^ | 2.08^***^ | 1.81^***^ | 2.54^***^ | 1.44^***^ | 1.67^***^ | 1.52^***^ |
|  | (0.09) | (0.31) | (0.10) | (0.23) | (0.28) | (0.09) | (0.33) | (0.30) | (0.40) |
| *N* | 620 | 617 | 620 | 616 | 584 | 620 | 577 | 613 | 577 |
| *R*^2^ | 0.08 | 0.06 | 0.00 | 0.03 | 0.02 | 0.01 | 0.17 | 0.19 | 0.19 |

Notes: Weighted OLS regression with clustered standard errors by municipality in parentheses; ***p<0.001, **p<0.01, *p<0.05, ^p<0.10

Table S5d.i: The Relationship between Decentralization by Organization Type and Decision Autonomy Reported by Frontline Health Workers (*Full Framework Model, Close Actor Accountability, Demographic Controls*)

| *Decision Autonomy* | (1)  Decen. Percep. | (2)  Autonomy  Plan. | (3)  Autonomy  Fin. | (4)  Autonomy  HR. | (5)  Autonomy Organiz. |
| --- | --- | --- | --- | --- | --- |
| *Administrative Form* |  |  |  |  |  |
| Decent-MUNI (ref. cent.) | 0.76* | -0.42** | -0.31* | -0.64** | -0.67** |
|  | (0.30) | (0.15) | (0.14) | (0.17) | (0.16) |
| Decent-ASSN (ref. cent.) | 1.05** | -0.35* | -0.26* | -0.25 | -0.18 |
|  | (0.26) | (0.15) | (0.13) | (0.18) | (0.11) |
| Decent-NGO (ref. cent.) | 0.69* | -0.39* | 0.17 | -0.37^ | -0.39** |
|  | (0.27) | (0.19) | (0.14) | (0.20) | (0.13) |
| *Capacity* |  |  |  |  |  |
| Years in Health Sector | -0.01 | 0.01 | 0.01 | 0.01 | 0.01 |
|  | (0.01) | (0.01) | (0.01) | (0.01) | (0.01) |
| Perceived Staff Leadership | 0.06 | 0.20* | 0.09 | 0.04 | 0.17** |
|  | (0.14) | (0.09) | (0.10) | (0.09) | (0.08) |
| *Accountability* |  |  |  |  |  |
| Evaluation Visits | -0.01 | -0.01 | -0.02* | -0.01 | 0.01 |
|  | (0.01) | (0.01) | (0.01) | (0.01) | (0.01) |
| Support Visits | 0.01 | 0.01 | 0.02** | 0.02 | 0.00 |
|  | (0.01) | (0.01) | (0.01) | (0.01) | (0.01) |
| *Resources* |  |  |  |  |  |
| Perceived HC Resources | 0.23* | 0.18** | 0.03 | 0.30** | 0.09** |
|  | (0.11) | (0.05) | (0.04) | (0.05) | (0.05) |
| *Additional Controls* |  |  |  |  |  |
| Age | 0.00 | -0.01 | -0.00 | -0.00 | -0.00 |
|  | (0.01) | (0.01) | (0.01) | (0.01) | (0.00) |
| Female | -0.04 | -0.04 | -0.14 | -0.04 | -0.03 |
|  | (0.12) | (0.08) | (0.10) | (0.08) | (0.08) |
| Education | 0.00 | 0.07 | -0.00 | 0.10^ | 0.05 |
|  | (0.07) | (0.06) | (0.04) | (0.06) | (0.04) |
| Constant | 2.07** | 0.93* | 1.32** | 0.57 | 1.38** |
|  | (0.62) | (0.38) | (0.36) | (0.43) | (0.30) |
| *N* | 522 | 576 | 569 | 577 | 579 |
| *R*^2^ | 0.19 | 0.16 | 0.13 | 0.23 | 0.17 |

Notes: Weighted OLS regression, binary indicator of decentralization, demographic controls models with clustered standard errors by municipality in parentheses; ^p<0.10, *p<0.05, **p<0.01

Table S5d.ii: The Relationship between Decentralization by Organization Type and Decision Autonomy Reported by Frontline Health Workers (*Full Framework Model, Close Actor Accountability, Role Type Controls*)

| *Decision Autonomy* | (1)  Decen. Percep. | (2)  Autonomy  Plan. | (3)  Autonomy  Fin. | (4)  Autonomy  HR. | (5)  Autonomy Organiz. |
| --- | --- | --- | --- | --- | --- |
| *Administrative Form* |  |  |  |  |  |
| Decent-MUNI (ref. cent.) | 0.80** | -0.43** | -0.23^ | -0.61** | -0.69** |
|  | (0.29) | (0.16) | (0.13) | (0.18) | (0.17) |
| Decent-ASSN (ref. cent.) | 1.10** | -0.37* | -0.21^ | -0.21 | -0.22^ |
|  | (0.25) | (0.15) | (0.11) | (0.19) | (0.11) |
| Decent-NGO (ref. cent.) | 0.72** | -0.40* | 0.24^ | -0.35^ | -0.40** |
|  | (0.26) | (0.19) | (0.13) | (0.20) | (0.13) |
| *Capacity* |  |  |  |  |  |
| Years in Health Sector | -0.01 | 0.01 | 0.01 | 0.01 | 0.01 |
|  | (0.01) | (0.01) | (0.01) | (0.01) | (0.01) |
| Perceived Staff Leadership | 0.07 | 0.20* | 0.09 | 0.05 | 0.16* |
|  | (0.11) | (0.09) | (0.07) | (0.06) | (0.07) |
| *Accountability* |  |  |  |  |  |
| Evaluation Visits | -0.01 | -0.01 | -0.02* | -0.01 | 0.01 |
|  | (0.01) | (0.01) | (0.01) | (0.01) | (0.01) |
| Support Visits | 0.01 | 0.01 | 0.02** | 0.02 | 0.00 |
|  | (0.01) | (0.01) | (0.01) | (0.01) | (0.01) |
| *Resources* |  |  |  |  |  |
| Perceived HC Resources | 0.22* | 0.19** | 0.01 | 0.29** | 0.10* |
|  | (0.10) | (0.05) | (0.04) | (0.05) | (0.05) |
| *Additional Controls* |  |  |  |  |  |
| Age | 0.00 | -0.01 | -0.00 | -0.00 | 0.00 |
|  | (0.01) | (0.01) | (0.01) | (0.01) | (0.00) |
| Female | -0.02 | -0.06 | -0.26** | -0.02 | -0.08 |
|  | (0.13) | (0.09) | (0.09) | (0.09) | (0.09) |
| Education | 0.08 | 0.02 | 0.05 | 0.14 | 0.01 |
|  | (0.09) | (0.07) | (0.05) | (0.09) | (0.05) |
| Nurse (ref. doctor) | 0.12 | -0.12 | 0.23* | 0.02 | 0.04 |
|  | (0.19) | (0.16) | (0.10) | (0.17) | (0.11) |
| Social Worker (ref. doctor) | 0.09 | -0.16 | -0.10 | 0.05 | -0.07 |
|  | (0.17) | (0.16) | (0.13) | (0.15) | (0.14) |
| Other Staff (ref. doctor) | 0.48 | -0.26 | 0.30 | 0.27 | -0.25^ |
|  | (0.32) | (0.18) | (0.19) | (0.25) | (0.13) |
| Constant | 1.67* | 1.28** | 1.10** | 0.33 | 1.59** |
|  | (0.80) | (0.47) | (0.40) | (0.52) | (0.38) |
| *N* | 522 | 576 | 569 | 577 | 579 |
| *R*^2^ | 0.20 | 0.16 | 0.16 | 0.24 | 0.18 |

Notes: Weighted OLS regression, binary indicator of decentralization, demographic controls models with clustered standard errors by municipality in parentheses; ^p<0.10, *p<0.05, **p<0.01

Inclusivity in global research

PLOS’ policy on inclusivity in global research aims to improve transparency in the reporting of research performed outside of researchers’ own country or community and ensures that PLOS publications reporting global research adhere to high standards for research ethics and authorship. Authors of relevant research articles may be asked to complete the questionnaire below, which outlines ethical, cultural, and scientific considerations specific to inclusivity in global research. This questionnaire may be requested when researchers have travelled to a different country to conduct research, if research uses samples collected in another country, research with Indigenous populations or their lands, or if research is on cultural artefacts. Researchers travelling to another country solely to use laboratory equipment will not normally be required to complete the questionnaire. However, the questionnaire can be requested at the journal’s discretion for any submission – if you have been requested to complete this questionnaire by the PLOS journal you submitted to, please do so.

Please complete the questionnaire below and include this as a Supporting Information file with your manuscript. Note that if your paper is accepted for publication, this checklist will be published with your article in the supporting information files. Please ensure that you reference the checklist in the main body of your manuscript. We suggest adding a subsection ‘Inclusivity in global research’ to your Methods section and adding the following sentence: “Additional information regarding the ethical, cultural, and scientific considerations specific to inclusivity in global research is included in the Supporting Information (SX Checklist)”

The questions have been designed to be applicable to a wide range of study types, and there are subsections for both human subjects research and non-human subjects research. If any of the questions are not relevant to your research please mark them as “N/A” as appropriate.

**Ethical considerations, permits and authorship**

*This section is applicable to all research types.*

Provide details as to who granted permissions and/or consent for the study to take place in the Methods section of your manuscript. This should include the names of **all** ethics boards, governmental organizations, community leaders or other bodies that provided approval for the study. If individuals provided approval refer to these people by their role or title but do not list their name(s).

Reported under “Sample of municipalities and health workers” in the “Empirical Strategy” section of the paper (around p. 15 of the unformatted version of the manuscript).

If there were any deviations from the study protocol after approval was obtained please provide details of these changes in the Methods section of your manuscript.

None.

Did this study involve local collaborators that are residents of the country where the research was conducted or members of the community studied? If you do not have any authors from said communities, please provide an explanation for this below.

As an external evaluation study of a political, policy reform, it would not be appropriate to include local collaborators as part of the authorship team.

Everyone listed as an author should meet PLOS’ criteria for authorship and all individuals who meet these criteria should be included in the author byline, rather than the acknowledgements. For further information please see the journal’s Authorship Policy.

**Human subjects research (e.g. health research, medical research, cross-cultural psychology)**

Did you obtain written informed consent from a representative of the local community or region before the research took place? How did you establish who speaks for the community? Details of written informed consent obtained from study participants should be reported separately in the Methods section of your manuscript.

IRB review at the University of Colorado Boulder and local review through the Honduran Ministry of Health determined that verbal consent was appropriate for this study context and population. Details of sample selection procedures are included in the manuscript – see pgs. 13-16 of the unformatted manuscript – and in the research design paper published prior to the present analysis by the group that designed the study (see Zarychta et al. 2019ab).

How did members of the local community provide input on the aims of the research investigation, its methodology, and its anticipated outcome(s)?

The research team designing the study received input from policymakers in the Ministry of Health, regional administrators, and local health center staff during the study design and local review process prior to data collection.

When engaging with the local community, how did you ensure that the informed consent documents and other materials could be understood by local stakeholders?

Part of the rationale for verbal consent was to ensure understanding; researcher contract an in-country organization with extensive experience to manage data collection efforts, and conducted extensive training with field staff on administering the verbal consent procedures.

Will the findings of the research be made available in an understandable format to stakeholders in the community where the study was conducted (e.g. via a presentation, summary report, copies of publications, etc.)? Please provide details of how this will be achieved.

The research team has already conducted outreach to Honduran Ministry of Health officials around the results of its studies, both in-person presentations and written documents in Spanish, and plans to continue doing so into the future to the extent it is welcomed.

**Non-human subjects research using specimens/ animals collected as part of the study, or those housed in archival collections. Examples include archaeology, paleontology, botany and zoology.**

Did the permission you obtained from a local authority to perform the study include an agreement on access to outputs and benefit sharing? This may include procedures to enable fair distribution of the benefits and resources arising from the research performed. Please include any details of Prior Informed Consent and Benefit Sharing Agreements obtained. These may be required by field-specific regulations, for example the Convention on Biological Diversity (CBD) and the associated Nagoya Protocol.

NA

If the material used in your study was imported, please A) provide the year it was imported and B) indicate whether permits were obtained to import/export the materials used, C) provide details of any permits obtained. If this information is not available, please indicate this.

NA

If you used archival specimens, please state how the material used in your study was acquired by the institute it is held in and provide details of any permits obtained for the original excavations/ sample collection. If this information is not available, please indicate this.

NA

How was the potential cultural significance of the materials collected in your study to local communities considered in your research design? Were Indigenous peoples and/or local researchers and institutions involved with archaeological excavations / collection of specimens? If so, please provide a description of their involvement.

NA

If your manuscript includes photographs of human remains please indicate whether authors obtained permission from descendants or affiliated cultural communities to do so.

NA
